# Temperature mediates biodiversity and metabolism of culturable lignocellulose-degrading consortia from intertidal wetlands

**DOI:** 10.1093/ismejo/wraf218

**Published:** 2025-10-04

**Authors:** Jiyu Chen, Min Yang, Qichao Tu, Lu Lin

**Affiliations:** Institute of Marine Science and Technology, Shandong University, Qingdao, Shandong 266237, China; Shandong Key Laboratory of Intelligent Marine Engineering Geology, Environment and Equipment, Qingdao 266237, China; Institute of Marine Science and Technology, Shandong University, Qingdao, Shandong 266237, China; Shandong Key Laboratory of Intelligent Marine Engineering Geology, Environment and Equipment, Qingdao 266237, China; Institute of Marine Science and Technology, Shandong University, Qingdao, Shandong 266237, China; Shandong Key Laboratory of Intelligent Marine Engineering Geology, Environment and Equipment, Qingdao 266237, China; Institute of Marine Science and Technology, Shandong University, Qingdao, Shandong 266237, China; Shandong Key Laboratory of Intelligent Marine Engineering Geology, Environment and Equipment, Qingdao 266237, China

**Keywords:** Chinese coasts, lignocellulose degradation, culturable bacteria communities, environmental temperature

## Abstract

Coastal bacteria play an important role in the conversion of terrestrial organic carbon (TerrOC). However, their ecological patterns and drivers remains elusive. Here, 180 bacterial communities from 10 regions along the Chinese coastline, covering an 18 000 km transect between 18.27°N and 39.82°N, were cultured under three typical lignocellulosic substrates, hardwood (aspen), softwood (pine), and herbaceous (rice straw), respectively. All the consortia showed a broad spectrum of TerrOC utilization, and displayed degradation capacities comparable with those previously established though preliminary in situ lignocellulose enrichment. Moreover, following the metabolic theory of ecology, annual average temperature of the sites stimulated community metabolism, even though all were cultured at 30°C. Consortia enriched on aspen exhibited the highest temperature sensitivity. 16S rRNA gene amplicon and metatranscriptomic sequencing analyses revealed temperature-dependent latitudinal diversity gradients, displaying a trend that was opposite of the temperature-diversity positive relationship observed in terrestrial lignin-degrading microbes. The community composition shifted to adapt to rising environmental temperature. To enhance lignin degradation, aspen consortia from high annual average temperature employed metabolic generalists, which induced expression of *dypB* centered gene families for lignin depolymerization and versatile pathways for degradation of lignin derivates. This study reveals the intrinsic drivers for coastal cultured lignocellulose degrading bacterial communities from an ecological perspective and deepens our understanding of the metabolic mechanisms in coastal TerrOC conversion.

## Introduction

Annually, substantial amounts (5 x 10^14^ g) of terrestrial organic carbon (TerrOC) are discharged into marine environments by rivers [1]. However, the residence time of most TerrOC in the ocean is short [2], suggesting that TerrOC degradation is likely active in coastal regions. Intertidal wetlands are critical ecological zones connecting terrestrial and marine ecosystems. They have strong carbon sequestration capacity and play an essential role in the complex processes of carbon cycling [3]. Our previous study cultured lignocellulose degrading bacterial consortia from an intertidal wetland of the East China Sea, revealing their roles in the degradation of various forms of terrestrial lignocellulose [4]. These consortia were derived from in situ lignocellulose enrichment samples from a single coastal zone site (29.95°N, 122.10°E) [5]. To further investigate the ubiquity of intertidal lignocellulose degrading consortia, the culturing of lignocellulose degrading bacterial consortia from multiple sites along the Chinese coasts, without preliminary in situ enrichment, may provide more detailed insights.

For a comprehensive understanding of the biogeographic pattern and intrinsic drivers for microbial communities, it is essential to investigate their metabolic capabilities. Environmental temperature directly accelerates the metabolic processes of consortia, and, thus, has been shown to be an important predictor [6]. The metabolic theory of ecology (MTE) predicts that the metabolism and growth rate of a community exponentially increases with the environmental temperature [7]. In addition, microbial diversity greatly affects community metabolic activity (e.g. carbon decomposition and sequestration) [8]. A strong association between diversity and metabolism in soil ecosystems has been observed [9–11]. Furthermore, ecological theory proposes that diversity increases with increasing environmental temperature, due to the kinetics of biological processes, and is termed “the Red Queen runs faster when she is hot” [12]. This positive temperature-diversity relationship has been observed for bacterial communities in forests, especially for lignin-degrading microbes [13, 14], although exceptions have also been reported [15, 16]. Therefore, a strong association among temperature, diversity, and metabolism has been revealed in terrestrial ecosystems [16, 17]. However, very little is currently known about the effect of environmental temperature on shaping the metabolism, diversity, and composition of cultured intertidal bacterial communities, which suffer from environmental disturbances, for TerrOC decomposition. Moreover, MTE provides a powerful framework for predicting the effects of temperature on community diversity and metabolism [18]. It remains unclear whether MTE can be applied to these consortia, when they are cultivated under uniform laboratory conditions.

TerrOC decomposition is a highly complex process [19]. Microbial communities derived from separate sources commonly display substantial differences in TerrOC decomposition, but our understanding of the underlying biological mechanisms remains limited. The effect is largely dependent on environmental heterogeneity and microbial community activity [19, 20]. Lignocellulose, as an abundant TerrOC, is composed of lignin, cellulose, and hemicellulose. Lignin exhibits a heterogenous and recalcitrant nature. Its content percentage and composition vary substantially with different plant types (e.g. hardwood, softwood, and herbaceous plants) [21]. Intertidal ecosystems are highly disturbed, characterized by strong dynamic interactions (e.g. tides and river runoff). Also, metabolic activity is associated with generalists and specialists within the microbial community. Habitat generalists have large genomes and, thus, can adapt to a broader range of niches. In contrast, specialists harbour small genomes, with specific environmental fitness [22]. In disturbed ecosystems, generalists play an important role in maintaining community stability by dampening microbial biogeographical patterns (e.g. a flatter slope of the distance–decay relationship) [23, 24]. However, it remains unresolved whether generalists and specialists in cultured intertidal consortia exhibit these reported patterns. Moreover, habitat generalists are commonly less competitive under optimal growth conditions. Conversely, specialists become dominant within a smaller niche space, reflecting a trade-off between local dominance and ecological versatility [25]. Our previous study suggested generalists in in situ intertidal lignocellulose degrading consortia showed greater genomic potential for lignin degradation [26]. However, there is the question of who would enhance lignocellulose degradation, especially the degradation of lignin, in cultured intertidal consortia? Under uniform culture conditions, would generalists, with their higher metabolic flexibility, outcompete specialists with specific fitness?

In this study, we cultured 180 lignocellulose degrading bacterial consortia, using different lignocellulosic substrates, from Chinese intertidal wetlands along an 18 000 km transect between 18.27°N and 39.82°N. The biogeographic patterns of intertidal community diversity and activity were investigated. Annual average temperature was identified as an important driver, where a temperature-dependent latitudinal diversity pattern was observed in the culturable consortia. MTE-based kinetic models provided powerful tools to further explore the effect of temperature on biodiversity and metabolism of the cultured lignocellulose degrading consortia. In contrast to the diversity dependent carbon decomposition of soil microbial communities, intertidal cultured consortia from higher environmental temperatures displayed significantly decreased taxonomic diversity and employed generalists with higher metabolic flexibility to enhance lignin degradation, providing a new perspective on coastal TerrOC conversion.

## Materials and methods

### Coastal sample collection and cultivation

The 18 000 km transect of Chinese coastline, from 18.27°N to 39.82°N, included tropical, subtropical, and temperate zones [27]. Coastal intertidal wetlands were selected as the inoculation sources to investigate coastal lignocellulose degrading bacterial consortia. The samples were collected from ten sites, from the southmost (Sanya, starting in early April 2021) to the northmost (Dandong, ending in early June 2021, Fig. 1A, Table S1, and Text S1). Six samples were collected at each site and immediately placed in an ice box for transport to the laboratory (Text S1). The samples were stored at 4°C for the subsequent culture experiments.

**Figure 1:**
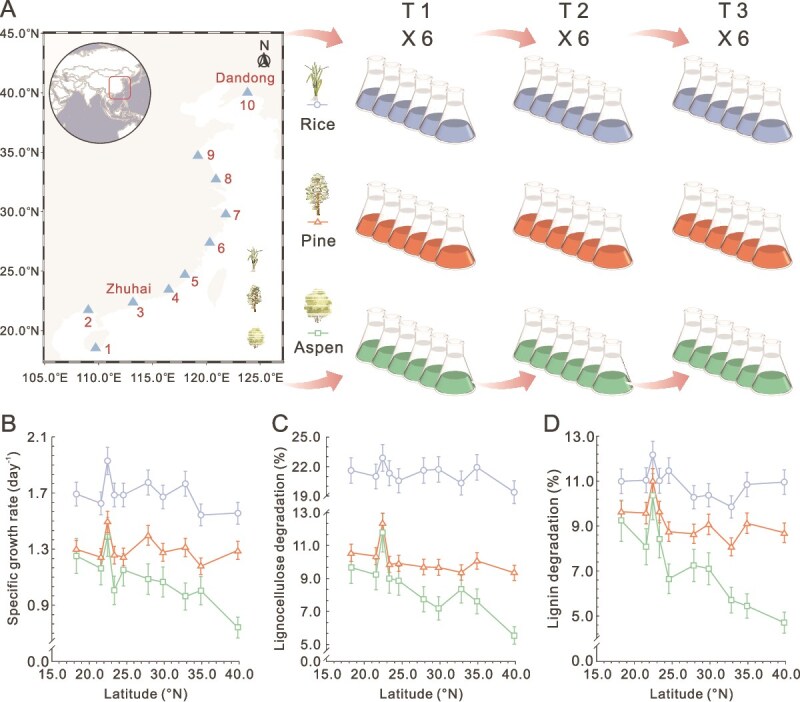
Study design for cultured lignocellulose degrading bacterial communities. (A) Schematic of the study design illustrating sampling from ten sites along the coastline of China, spanning from the southmost (Sanya) to the northmost (Dandong). A total of 60 mudflat intertidal sediment samples were collected and cultured in MB medium at 30°C, supplemented with 3% of the corresponding lignocellulose substrate (aspen, pine, and rice straw). T: transfer. (B) Specific growth rates of the bacterial consortia at T3 enriched on different lignocellulose substrates along the coastal latitudinal gradient. (C-D) Lignocellulose (C) and lignin (D) degradation of the bacterial consortia (T3) enriched on the respective lignocellulose substrates. Data are presented as mean values ± standard deviation, n = 6 biological replicates.

Each sample was incubated in MB medium at 30°C, 150 rpm for 7 days, as transfer 1 (T1). From T1 to T3, 5 ml of culture suspension at day 5 was successively transferred to 100 ml fresh MB medium. 16S rRNA gene copy number was measured to monitor the growth of bacterial communities. The culture experiments were performed with six biological replicates. The details are described in Supplementary Text S1.

### Substrate measurement and analysis

The consumption of lignocellulosic substrate was measured by weighing method [28]. The amounts of cellulose, hemicellulose, and lignin were examined by the Laboratory Analysis Protocol of the National Renewable Energy Laboratory, Golden, CO, USA [29]. The soluble lignin derivates were analyzed by gas chromatography–mass spectrometry (GCMS-QP2020 NX, Shimadu, Kyoto, Japan) [30]. The experiments were performed with three biological replicates. The details are provided in Supplementary Text S1.

### Extracellular enzyme assays

Enzymatic activity assays were monitored by a Cytation 5 microplate reader (BioTek, Vermont, USA) at 25°C. Culture supernatant was collected by centrifugation (12 000 g, 10 min, 30°C) as the crude enzyme extract. The activity of glucanase, xylanase, β-glucosidase, and β-xylosidase were measured, respectively (Text S1). The experiments were performed with three biological replicates.

### High-throughput DNA sequencing and data analysis

Three samples at T3 were randomly selected from each sampling site for 16S rRNA gene amplicon sequencing. Additionally, aspen consortia, from Zhuhai (ZA) and Dandong (DA), with three biological replicates, were collected at Day 5 of T3 for metatranscriptome sequencing (Text S1). The metagenomic data for corresponding in situ intertidal samples (10 sites) were retrieved from the NCBI SRA database (PRJNA957716) [31]. DADA2 R package (v1.3.2) was used for the 16S rRNA gene sequencing data analysis (Table S2). Trimmomatic v0.39 software [32], Trinity v2.8.5 software [33] and MEGAHIT (v1.2.9) were employed for metatranscriptomic and metagenomic sequencing data analysis, respectively. The carbohydrate-active enzymes (CAZy) database (http://www.cazy.org; CAZy update, April 2023) [34] and lignin catabolism database [35] were used for the lignocellulose degrading functional profiling analysis. Null mode analysis (iCAMP) was performed by the NST package (v3.1.10) in R (v4.2.1). All statistical analyses were performed by R (v4.2.1). Shannon index, specific growth rate, lignin degradation, and lignocellulose degradation were used to the linear model of MTE, $\ln \left(\mathrm{R}\right)=a-{E}_a\times \frac{1}{KT}$ [14]. The details are provided in Supplementary Text S1.

## Results

### Low latitude and high temperature promote community metabolism

In this study, 180 consortia were cultured from 10 inoculation sites with 3 substrates (Fig. 1A). After three transfers, all consortia showed stable growth in MB medium at 30°C when supplemented with 3% lignocellulose substrate, reaching 10^8^–10^9^ copies/ml (Fig. S1A). The growth rates increased in the order aspen < pine < rice (Fig. 1B). Similarly, consortia enriched on rice showed the highest lignocellulose degradation, followed by consortia enriched on pine and aspen, respectively (Fig. 1C and D and Fig. S1B and C). Together with the widespread lignocellulose degrading gene families (Fig. S2), this revealed the feasibility of extensive culturable lignocellulose degrading bacterial consortia in the Chinese coastal environments, as well as a substrate dependent growth and degradation gradient.

**Figure 2:**
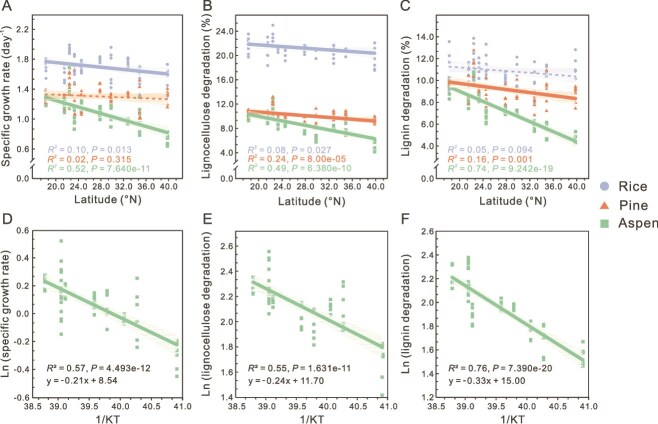
Relationships between community metabolism and environmental variables. (A-C) Scatterplots of latitude vs. specific growth rate (A), lignocellulose degradation (B) and lignin degradation (C). (D-F) Scatterplots of temperature vs. specific growth rate (D), lignocellulose degradation (E), and lignin degradation (F) for aspen degrading consortia. The natural log of specific growth rate, lignocellulose degradation, and lignin degradation values were used for analyzing the relationships between community metabolism and temperature, which was expressed as the inverse of the annual average temperature in degrees Kelvin. Each line represents a least squares regression fit and the shaded area represents the 95% confidence interval, n = 6 biological replicates.

The metabolic capacities of the consortia, as indicated by growth rate and substrate degradation, exhibited temperature dependent latitudinal variations. Consortia enriched on rice lignocellulose showed negative correlation between growth rate and latitude (Fig. 2A). The highest growth rate was from the southern Zhuhai site (22.44°N, 24.00°C), at 1.24-fold (*P* = 4.48e-05) higher than the lowest consortia from the northern Lianyungang site (34.89°N, 15.00°C, Fig. 1B). The negative correlation became stronger when consortia were enriched on aspen (Fig. 2A). The highest growth rate of aspen consortia in the south (Zhuhai) was 1.87-fold (*P* = 9.49e-07) higher than in the north (Dandong, 18.27°N, 10.50°C, Fig. 1B). Pine and aspen degradation also exhibited negative correlations with latitude, especially for aspen lignin degradation (Fig. 2B and C). The degradation of aspen lignin and lignocellulose at Zhuhai were both ~2.2-fold higher than consortia at Dandong (*P* = 1.83e-10 and 2.49e-07, Fig. 1C and D). Coinciding with the latitude decay, environmental temperature stimulated community metabolism (Fig. S3A-C). Overall, high temperature and low latitude, stimulated community growth and accelerated lignocellulose degradation, especially for aspen consortia.

As temperature accelerates metabolic processes, we further applied MTE directly to the metabolic data of the consortia enriched on herbaceous and woody lignocellulose. Boltzmann-Arrhenius plots were used to assess the metabolic dependence of the metabolic processes on temperature. The statistical analyses identified the strongest Boltzmann exponential relationships as those between the log-transformed lignin degradation and the reciprocal temperature (1/KT) for aspen consortia, followed by growth rate and lignocellulose degradation (Fig. 2D-F). Temperature explained 54.6%–76.4% (*R*^2^ values) of the variations in metabolism of aspen consortia. Moreover, the estimated activation energies (*E_a_*, the slope of plots with sign reversed) of aspen consortia were the highest among the three different substrates, highlighting the exponential effects of temperature on consortia enriched on aspen (Fig. 2D-F and Fig. S3D-I). In contrast, pine and rice consortia showed a lower dependence on temperature. Especially, the correlations were not significant for lignin degradation of rice consortia and growth rate of pine consortia (Fig. S3D-I). In summary, temperature dependent metabolism was observed in the culturable consortia, and it varied from substrate to substrate.

### Temperature-dependent latitudinal diversity patterns for culturable coastal lignocellulose degrading consortia

**Figure 3:**
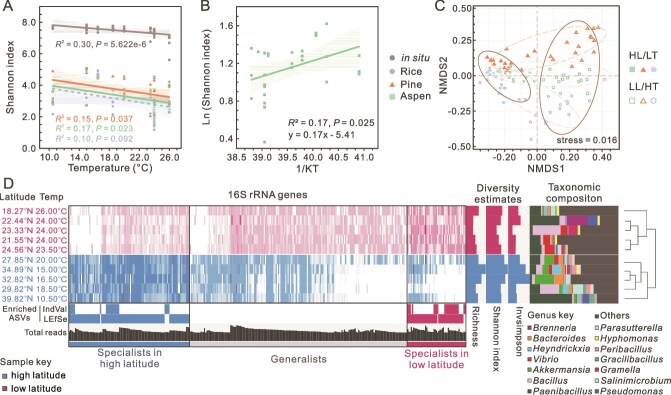
Changes of the community diversity and composition along temperature-dependent latitudinal gradient in Chinese coasts. (A) The scatterplot of community alpha diversity vs. annual average temperature of the source sites. (B) The relationship between community diversity and environmental temperature for aspen degrading consortia, which was expressed as the inverse of annual average temperature in degrees Kelvin. The natural log of Shannon index values was used to indicate community diversity. (C) A nonmetric multidimensional scaling (NMDS) profile of the cultured consortia based on the Bray-Curtis dissimilarities. Ellipses indicate the 95% CI (confidence interval) grouping effects of substrate and temperature/latitude. HL: high latitude, LT: low temperature, LL: low latitude, HT: high temperature. (D) Temperature/latitude separated cultured bacterial consortia, determined by 16S rRNA gene amplicon sequencing. The vertical lines in the left plot represent ASVs, where intensities indicate the log-normalized abundances at low and high latitude. The bars below indicate ASVs that were significantly enriched in the sampling sites as determined by either indicator species analysis (IndVal) or linear discriminant analysis effect size (LEfSe). Diversity estimates charts show the richness, Shannon index, and Inverse-Simpson index. Taxonomic profiles show community composition of major genera (> 1%). The hierarchical clustering of samples is based on Ward’s method. Data (A-D) are from three biological replicates for cultured consortia and six biological replicates for in situ consortia.

High throughput sequencing analysis was performed to examine the relationships between taxonomic diversity and temperature/latitude (Table S2). We observed Shannon index values increased with latitudinal gradient from 18.27°N to 39.82°N, and decreased with annual average temperature, from 10°C to 26°C for in situ and cultured lignocellulose degrading consortia (Fig. 3A and Fig. S4A). For cultured consortia, the correlation was significant for wood degrading consortia, especially the aspen consortia (Fig. S5A and B). This demonstrated warming decreased community diversity, especially for consortia enriched on woody substrates. Moreover, we applied MTE to assess whether the community diversity also followed the exponential effects of temperature. Boltzmann-Arrhenius plots of the natural logarithm of diversity, as indicated by Shannon index, showed a linear correlation of inverse absolute temperature, confirming temperature dependence (Fig. 3B and Fig. S4B-D). The estimated *E*_a_ of cultured consortia was higher than that of *in situ* lignocellulose degrading consortia, although rice consortia showed a non-significant relationship. It was further increased in the order pine < aspen and reached the maximal values in aspen consortia for the taxa that expressed lignocellulose/lignin degrading genes (Fig. S5C-D). This indicated that the community diversity of aspen degraders showed the highest temperature sensitivity.

To further verify the importance of environmental factors, null model was employed to analyze the relative importance of deterministic and stochastic processes in structuring the cultured consortia enriched on the three substrates. Deterministic processes contributed over 50% relative importance of community variation, suggesting that taxonomic compositions were relatively deterministic (Fig. S6). Consequently, the impact of key environmental factors (substrate and latitude/ temperature) in community composition was evaluated by redundancy analysis. Significant correlations between the factors and consortia were observed (*P* < 0.01, Fig. S4E). Lignocellulose substrate was the primary factor determining coastal lignocellulosic degrading consortia (*P* = 0.001, relative importance (RI) = 43.1%). This was supported by nonmetric multidimensional scaling (NMDS) analysis, where a separation of consortia by substrate was observed across the ten coastal zones in China (Fig. 3C, Fig. S7, Table S3, and Text S2). In addition, NMDS also showed a latitudinal/temperature separation of consortia across all substrates (Fig. 3C and Table S3). The consortia were grouped into two clusters, i.e. consortia from high latitudes (27.85°N-39.82°N) and consortia from low latitudes (18.27°N-24.56°N). The latitudinal/temperature separation caused large shifts in community composition (Fig. 3D, Figs S8-S9, and Text S2).

### Cultured consortia display enhanced metabolic capacity as diversity decreased

**Figure 4:**
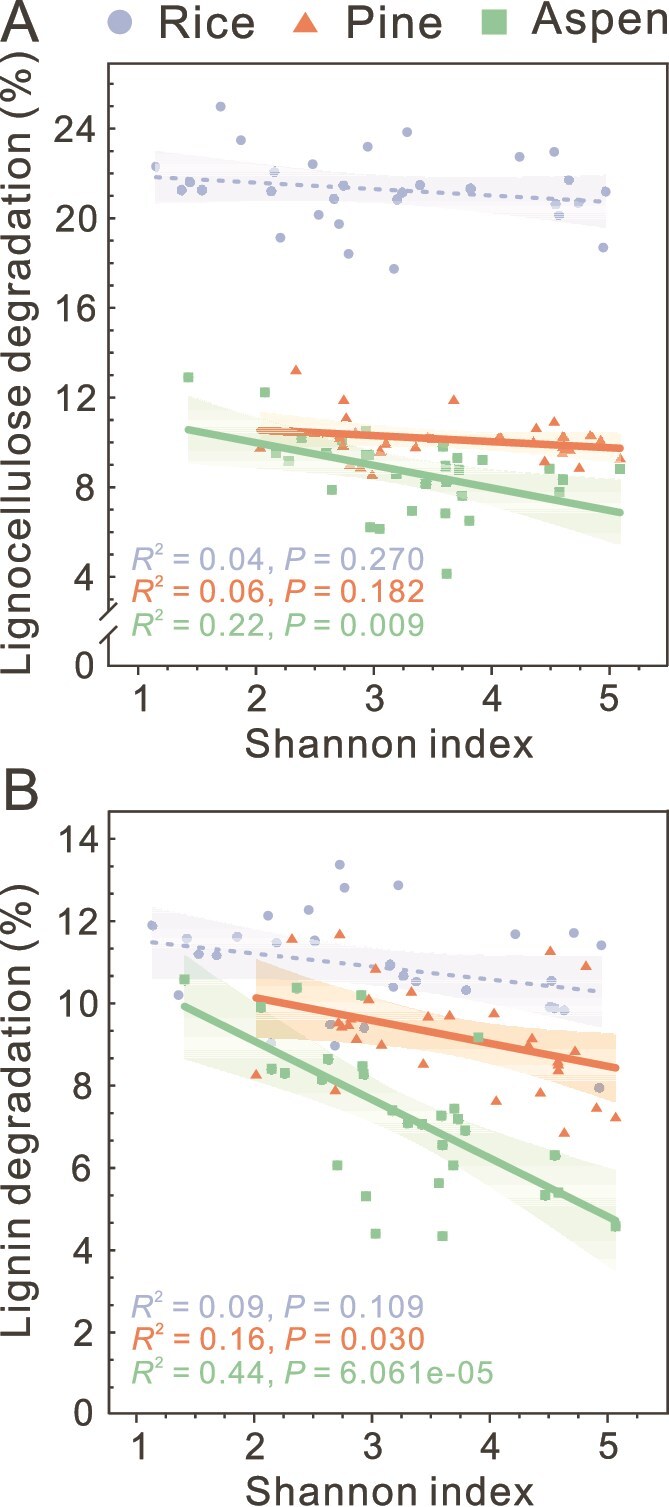
Relationships between community diversity and carbon decomposition. Scatterplots of Shannon index vs. lignocellulose degradation (A) and lignin degradation (B). Each line represents a least squares regression fit and the shaded zone represents the 95% confidence interval. Dashed lines indicate non-significant relationships. n = 3 biological replicates.

We further examined the relationship of community diversity and metabolism, both of which exhibited temperature sensitivity. Lignocellulose/lignin degradation decreased with increasing Shannon index values (Fig. 4). Moreover, the pattern was substrate dependent. When pine and aspen with higher lignin content (~25%) were employed, the correlation was significant (Fig. 4). Conversely, the correlation was not significant for rice (herbaceous, Fig. 4). Hence, consortia with reduced diversity exhibited a stronger capacity for woody lignin degradation.

**Figure 5:**
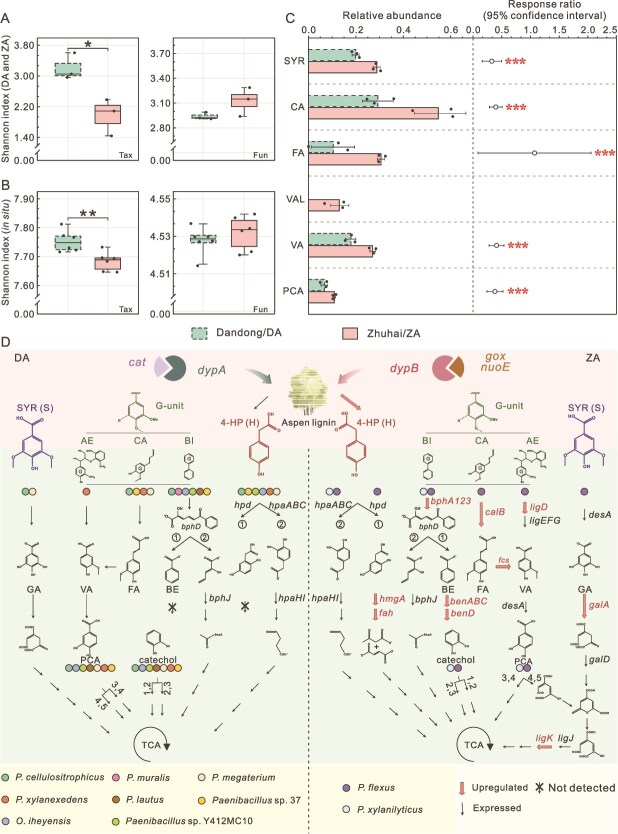
ZA consortia displayed enhanced lignin degradation, yet reduced diversity. (A-B) Alpha diversity of cultured lignocellulose degrading consortia at Zhuhai and Dandong (A) and in situ consortia (B) at the levels of taxonomy (Tax) and functional genes (Fun). “^*^”: *P* < 0.05, “^**^”: *P* < 0.01 using two-tailed Student’'s *t*-test. (C) The relative abundances and their response ratios for monomeric lignin-derived compounds generated by ZA and DA consortia (ZA vs. DA). “^***^”: *P* < 0.001 using the two-tailed Response Ratio test [36]. (D) The lignin degradation strategies employed by ZA and DA consortia. SYR: syringate, CA: coniferyl alcohol, FA: ferulic acid, VAL: vanillyl alcohol, VA: vanillic acid, PCA: protocatechuic acid, AE: aryl ether, BI: biphenyl, 4-HP: 4-hydroxyphenylacetic acid, GA: gallate, BE: benzoate. Upregulated: ≥ 2-fold change with *P* < 0.05. Data (A-C) are presented as mean values ± standard deviation. Data (A-D) are from three biological replicates for cultured consortia and six biological replicates for in situ consortia.

To explore the substrate degradation mechanism(s) of the consortia, two groups of aspen consortia, ZA and DA, at the two endpoints of the latitude/temperature gradient, were targeted for the metatranscriptome sequencing, as they exhibited the greatest differences in lignocellulose degradation and community diversity (Fig. 5A and Fig. 1C-D). ZA and DA both showed the decreased diversity at the taxonomic and functional levels, compared to *in situ* lignocellulose degrading consortia (Fig. 5B). Moreover, the diversity of functional genes was just slightly reduced by ~1.5-fold, in contrast to the substantial declined taxonomic diversity by 2.4–4.0-fold. A total of 254 gene families were found to be expressed during lignocellulose degradation. These were further divided into cellulose (47 gene families) and hemicellulose (59 gene families) hydrolysis, and lignin degradation (148 gene families, Table S5A). A similar pattern was observed for hemi−/cellulose hydrolysis, without substantial biological differences between ZA and DA consortia (1.15-fold change, Fig. S10 and Text S2).

In contrast to similar hemi−/cellulose hydrolysis, ZA and DA consortia exhibited significant differences in lignin degradation (2.2-fold change, Fig. 5C and D). ZA consortia mobilized a DypB based enzyme group for lignin depolymerization, where the *dypB* gene was significantly induced (Fig. 5D and Fig. S11A and B). The *poxB*, *gox*, and *nuoE* gene families were also observed to be upregulated in the ZA consortia (Fig. 5D and Fig. S11A and B). These auxiliary enzymes assist *dypB* during lignin depolymerization (Text S2). In contrast, DA consortia with their higher expression level of *dypA*, also highly expressed *cat* and *gpx* (Fig. 5D, Fig. S11A-B, and Text S2). Correspondingly, more lignin monomers were released in the samples of ZA consortia (Fig. 5C). The results demonstrated that the DypB based enzyme cocktail employed by ZA consortia had higher lignin depolymerization capacity than the DypA based enzyme group used by DA consortia.

In response to the greater abundance of lignin derivatives in ZA consortia, there were higher induced expression levels of genes involved in aromatic compound degradation pathways, especially pathways of S- and G-type lignin units (Fig. 5D and Fig. S11E). Aligning with the higher abundance of syringate in ZA consortia, the expression levels of *galA* and *ligK* in the syringate pathway were only observed or significantly upregulated compared to that of DA consortia (Fig. 5D and Fig. S11 C-D). Gene families (e.g. *bphA123*, *calB*, *fcs*, and *ligD*) involved in G-type unit degradation pathways were also induced in ZA consortia, which coincided with the more abundant coniferyl alcohol, ferulic acid, and vanillic acid (Fig. 5C and D). In addition, ZA consortia employed versatile pathways to degrade lignin derivatives (Fig. 5D). In contrast, DA consortia only expressed a pathway for a relevant lignin derivative. In ZA consortia, two biphenyl degradation pathways were actively expressed, of which the gene families *bphA123* and *benABCD* from pathway 1 were significantly up-regulated. Similarly, two 4-hydroxyphenylacetic acid degradation pathways were identified in ZA consortia, yet genes (e.g. *fah*) from pathway 1 were not expressed in DA consortia (Fig. 5D and Fig. S11D). Together, these aromatic compound degradation pathways were actively expressed by ZA consortia to catabolize the abundant lignin depolymerization products.

ZA consortia employed metabolic generalists with more than four metabolic pathways to induce the expression of lignin degrading genes. *Paenibacillus xylanilyticus* and *Priestia flexa* in ZA consortia were the dominant species to express lignin degrading genes, accounting for 47.5% and 43.0% in aspen lignin degraders, respectively (Fig. S12A). They both expressed genes involved in biphenyl, 4-hydroxyphenylacetic acid, and 3-hydroxycinnamic acid degradation pathways. Moreover, *P. flexa* also actively expressed genes in the syringate, aryl ether, and coniferyl aldehyde degradation pathways, exhibiting its metabolic flexibility. The relevant *Paenibacillus* (ASV4) and *Priestia* (ASV9) were also habitat generalists (Fig. S8), indicating that metabolic flexibility expanded the breath of their natural habitats. Conversely, only one metabolic generalist, *Paenibacillus cellulositrophicus*, was identified in DA consortia to be involved in 3-hydroxycinnamic acid, 4-hydroxyphenylacetic acid, biphenyl, coniferyl aldehyde, and syringate degradation pathways (Fig. 5). The corresponding *Paenibacillus* ASV8 was a habitat generalist. In contrast to the highly abundant generalists in ZA consortia, its abundance was only 24.3% in DA consortia (Fig. S12B). Therefore, DA consortia mobilized many more species to degrade such a complex polymer. These included *Oceanobacillus iheyensis*, *Paenibacillus* sp. Y412MC10, *Paenibacillus* sp. 37, *Paenibacillus xylanexedens*, *Paenibacillus lautus*, *Priestia megaterium*, and *Peribacillus muralis*, accounting for over 51% of lignin degraders (Fig. 5D and Fig. S12). They commonly exhibited a relatively narrower metabolic flexibility. *O. iheyensis* possessed only pathway 2 for degradation of 4-hydroxyphenylacetic acid and *Paenibacillus* sp. Y412MC10 only expressed pathway 2 for degradation of biphenyl (Fig. 5D). In addition, *P. xylanexedens* (ASV29) actively expressed genes for aryl ether and coniferyl aldehyde degradation, whereas *P. lautus* (ASV18) and *P. muralis* (ASV15) were involved in biphenyl degradation. These three members were habitat specialists from high latitudes, respectively (Fig. S8). This indicated that these metabolic specialists, with three or fewer pathways, also have a narrower niche. Overall, the results demonstrated that generalists within a community showed an advantage over specialists in lignin degradation.

## Discussion

### Coastal cultured bacterial consortia exhibit pervasive lignocellulose degradation capacities

In contrast to the numerous reports on terrestrial lignocellulose degrading consortia [37–39], only a few studies have investigated marine lignocellulose degrading microbial consortia, most of which utilized *in situ* lignocellulose enrichments to retrieve the relevant consortia [40]. This is because the turnover rates of TerrOC are likely underestimated in coastal ecosystems [1, 41]. Here, we directly cultured native coastal bacterial consortia, covering ten intertidal zones along the Chinese coast from 18.27°N ~ 38.82°N, using different lignocellulose substrates (Fig. 1). All cultured consortia exhibited the capacity to degrade hardwood (aspen), softwood (pine), and herbaceous lignocellulose (rice straw). It indicated that lignocellulose degrading consortia are widespread along the Chinese coastline, aligning with the ubiquitous lignocellulose degrading gene families (Fig. S2). To further evaluate their degradation capacity, we compared consortia from Wenzhou (27.85°N, 121.02°E) and Ningbo (29.82°N, 122.03°E), with our previous *in situ* enriched culturable consortia from a nearby site at Zhoushan (29.95°N, 122.10°E) [4]. In the previous study, 6-month *in situ* lignocellulose enrichments were performed at Zhoushan, with three substrates (aspen, pine, and rice straw), after which they were cultured as described in this study. The Wenzhou and Ningbo consortia, without any *in situ* enrichment treatment in this study, showed degradation capacities comparable with those of Zhoushan which had undergone a 6-month *in situ* enrichment, especially for lignin degradation (Table S6). This indicates that bacterial communities in intertidal zones play a role in TerrOC conversion, which exhibits the higher rate than we expected. The marine-derived degraders (e.g. *P. lautus*, *P. flexa*, *Rossellomorea marisflavi*, and *O. iheyensis*, which were reported to degrade various organic carbon polymers (i.e. lignin, cellulose, and chitin) [42, 43], were observed in the cultured consortia. Terrestrial-derived degraders (e.g. *P. xylanilyticus* and *P. cellulositrophicus* [44, 45]) were also detected in the consortia, possibly due to our enriched consortia at the land-sea interface. Furthermore, previously unrecognized lignin degraders were also observed (e.g. *Fictibacillus arsenicus* and *Rossellomorea vietnamensis*).

The cultured consortia, ZA and DA, showed varied compositions with *in situ* lignocellulose degrading consortia at Zhuhai and Dandong (Fig. S12), possibly due to the specific culture condition (i.e. 30°C, MB medium and aspen substrate) in this study. *P. flexa* and *P. xylanilyticus*, as the dominant lignin degraders in ZA (> 42%), occupied 0.003–0.006% in *in situ* consortia at Zhuhai, whereas *P. cellulositrophicus*, *Paenibacillus* sp. 37, *O. iheyensis*, *Paenibacillus* sp. Y412MC10, *P. lautus*, and *P. xylanexedens*, which were the enriched lignin degraders in DA (> 1%), accounted for 0.004%–0.01% in *in situ* samples at Dandong (Fig. S12). Conversely, the ~50% lignocellulose degraders, with high abundance (> 0.3%) in *in situ* consortia, were cultured in our study (e.g. *Sorangium cellulosum*, *Rhodopseudomonas palustris* in ZA consortia and *Ramlibacter tataouinensis*, *Desulfosarcina ovata*, and *Desulfosarcina alkanivorans* in DA consortia), even though they were rare species (0.0002%–0.004%, Fig. S13A). In contrast to the variable species, the lignin degrading pathways were relatively similar between the cultured and *in situ* consortia, with the exception of the phenol meta-cleavage pathway at Zhuhai and the degradation pathways for 4-hydroxyphenylacetic acid (pathway 1), diarylpropane, benzoate, and *p*-cumate degradation at Dandong that were not expressed in the relevant cultured consortia (Fig. S13B). It coincided with the current view that environment selects functional genes, rather than species [35]. Further investigating the TerrOC metabolic potential of *in situ* microbial consortia, in the near future, is essential to comprehensively understanding the ubiquity of lignocellulose degrading consortia in Chinese coastal zones.

### Annual average temperature affects the decomposition of recalcitrant lignin in cultured communities

The importance of environmental temperature in controlling community metabolic processes is well documented [16, 46]. However, little is known on how this affects cultured microbial communities. By examining 180 culturable consortia across an annual average temperature gradient (10.5°C–26.0°C), our results showed that environmental temperature of the source sites also had a pervasive influence on the metabolism of lignocellulose degrading bacterial communities, even though they were all cultured at 30°C. Metabolism is a complex biological process. *R* in MTE (*R* ∝ e^–E/KT^), hence, could be represented by the rates of these metabolic processes (e.g. substrate degradation, enzyme reactions, population growth, or respiration) [47]. Our study pinpoints that lignin degradation, rather than hemi−/cellulose degradation, could be well characterized by the exponential temperature dependent metabolism in cultured lignocellulose degrading bacterial consortia (Fig. 2). Moreover, the temperature dependence (*E_a_*) was negatively associated with substrate degradation. Aspen consortia showed the highest temperature dependence, yet the lowest lignin degradation, whereas rice consortia exhibited the opposite pattern. This could be related to the intrinsic temperature sensitivity of different organic carbon compounds [48]. The carbon quality temperature hypothesis proposes that less reactive and more recalcitrant organic compounds commonly have higher temperature sensitivities [49]. Among the three substrates in this study, aspen, as a hard wood, possesses a more recalcitrant nature due to its much higher lignin content, followed by pine (softwood) and rice straw (herbaceous, Table S4). Moreover, aspen lignin has a higher proportion of S-lignin units, which are more stable than H-lignin units [50]. Conversely, rice straw, as herbaceous lignocellulose, contains a greater amount of less recalcitrant hemi−/cellulose and a low concentration of lignin, with abundant H-type lignin units.

### Higher annual average temperature reduces community diversity and alters community composition

Temperature is also expected to have strong influence on community diversity [18]. This positive temperature-diversity relationship has been well documented in animal and plant species [51, 52] and also been successively observed for bacteria and fungi in forest soils, tundra, marine systems, and elsewhere [14, 53, 54]. Moreover, bacterial diversity commonly exhibits varied geographic patterns over latitudinal gradients. Besides decreased diversity with increasing latitude, a unimodal distribution along a latitudinal gradient has also been reported in the near-surface Atlantic Ocean and eastern China, with a maximal diversity around 40°N [55, 56]. In our study, the overall diversity of consortia also reached its highest diversity from inoculum retrieved at ~40°N. This could be explained by that intermediate temperatures (e.g. 10°C) might stimulate diversification of the bacterial community [56]. Therefore, the overall diversity decreased along the gradient of rising annual average temperature, even though warming increases metabolic activity. Furthermore, the negative relationship became stronger in aspen consortia when only lignocellulose/lignin degraders were examined. Similar results were recently revealed *in situ* soil warming experiments, where even though soil carbon degradation was greatly promoted under warming, the microbial diversity was lower [16, 17]. This was inconsistent with the current view that, in forest soils, carbon decomposition depends on the community diversity, with a greater species diverse allowing access to more pathways [57]. It indicates that stimulation of carbon decomposition at the warming site might be the result of changes among individual species, rather than an increase in overall diversity (Fig. S14). Thus, ZA and DA consortia, from either end of the temperature gradient, exhibited the greatest variations in taxon composition. Shifting to *Paenibacillus* and *Priestia* taxa in ZA consortia, with much more active and versatile lignin degrading pathways, demonstrated that the community shifted to taxa that readily persisted and increased in metabolic activity under warmer conditions (~30°C).

### Metabolic generalists became dominant lignin degraders in response to lower diversity

In contrast to the reduced taxonomic diversity under higher temperature, the functional genes participating in lignin degradation were actively expressed to accelerate its degradation, revealing several key bacterial functional gene groups. The *dypB* gene, coupled with *poxB*, *gox*, and *nuoE*, exhibited high efficiency for aspen lignin depolymerization. Versatile pathways were observed to actively catabolize lignin depolymerization products. These active pathways were driven by metabolic generalists. The generalists presented an increasing expression pattern with higher temperatures, including the relative expression abundances and metabolic flexibility, even though they exhibited the dampening biogeographical pattern (Fig. S15). *P. xylanilyticus* and *P. flexa* were rare generalists (0.25% and 0.03%) in DA consortia, yet increased to 43% and 47.5% in ZA consortia, respectively. *P. flexa* in ZA consortia exhibited a wide substrate utilization spectrum, including syringate, 4-hydroxyphenylacetic acid, biphenyl, aryl ether, coniferyl aldehyde, and 3-hydroxycinnamic acid. In contrast, the only enriched generalist, *P. cellulositrophicus*, in DA consortia exhibited a relatively narrower range of substrate utilization, lacking active pathways for aryl ether, biphenyl (pathway 1), and 4-hydroxyphenylacetic acid (pathway 1). Overall, generalists have advantages in heterogenous lignin degradation.

In conclusion, this study provides two important implications for understanding coastal TerrOC bioconversion. First, the annual average temperature has a pervasive influence on culturable lignocellulose degrading bacterial communities, including community diversity and activity. Higher annual average temperature intensified lignin degradation, which was associated with the degree of substrate recalcitrance. Lignin degradation should be incorporated into the MTE-based kinetic models, which could provide tools to project the effects of temperature on metabolism of intertidal cultured consortia. Second, this study deepens our understanding of TerrOC metabolic strategies, including the efficient enzyme groups and pathways. Because lignin degradation is primarily enhanced by accumulating generalists with higher metabolic flexibility, it provides inspiration for microbiome engineering through the sub-population ratio control of key bacterial generalists and specialists to regulate TerrOC decomposition.

## Supplementary Material

Supplementary_materials_10_13_final_wraf218

TableS1_wraf218

TableS2_wraf218

TableS5_wraf218

## Data Availability

The 16S rRNA gene amplicon and metatranscriptome sequencing raw data were deposited in the NCBI SRA database (PRJNA1029913). The metagenomic raw data for in situ intertidal samples, published previously [31], are available in the NCBI SRA database (PRJNA957716).
