# Supplementary material for: Temperature mediates biodiversity and metabolism of culturable lignocellulose-degrading consortia from intertidal wetlands
Source: ISME J. 2025 Oct 4;19(1):wraf218. doi: 10.1093/ismejo/wraf218 (PMC12551453; doi:10.1093/ismejo/wraf218)
Supplement: Supplementary_materials_10_13_final_wraf218 [file supplementary_materials_10_13_final_wraf218.pdf]

Supplementary Information for

Temperature mediates biodiversity and metabolism of culturable lignocellulose-degrading consortia from intertidal wetlands

Jiyu Chen[1, 2#], Min Yang[1, 2#], Qichao Tu[1, 2], Lu Lin[1, 2*]

1. Institute of Marine Science and Technology, Shandong University, Qingdao, China

2. Shandong Key Laboratory of Intelligent Marine Engineering Geology, Environment and Equipment, Qingdao 266237, Peoples R China

[#] These authors contributed equally to this work

* For correspondence: Lu Lin, Shandong University, Qingdao, Shandong, 266237, China, linlu2019@sdu.edu.cn

This PDF file includes:

Supplementary text 1 to 2

Supplementary Figure 1 to 15

Supplementary Table 1 to 6

SI Reference

Supplementary Text

Text S1 Materials and Methods

Coastal site description and sample collection

The sampling sites were away from riverine inputs to avoid potential freshwater disturbances, as previously described [1, 2]. For each location, six replicate samples were collected from 200 m intervals along a spatially explicit transect (400 m × 200 m). For each sample, five surface sediment cores, to a depth of ~15 cm, were collected from a randomly selected 1 x 1 m square (all four corners and the central point) on the ebb tide, mixed and immediately placed in an ice box for transport to the laboratory. The samples were stored at 4 ℃ for the subsequent culture experiments.

Cultivation experiments for lignocellulose degrading bacterial consortia

Each sample (~10 g) was resuspended in 100 mL 2216E solution (5 g Tryptone, 19.45 g NaCl, 1 g Yeast Extract, 0.1 g Ferric citrate, 12.78 g $MgCl_2 \cdot 6H_2O$, 3.24 g Na_2SO_4, 2.38 g $CaCl_2 \cdot 2H_2O$, 0.55 g KCl, 0.16 g Na_2CO_3, 0.08 g KBr, 0.034 g SrCl, 0.004 g $Na_2SiO_3 \cdot 9H_2O$, 0.022 g H_3BO_3, 0.0024 g NaF, 0.0016 g NH_4NO_3, and 0.008 g Na_2HPO_4 in 1 L ddH_2O) at 25℃, 150 rpm for 30 min [3]. Next, 5 mL of the suspension was washed with 5 mL MB medium and then transferred into 100 mL MB medium (0.33 g/L NH_4Cl, 0.88 g/L $NaNO_3$, 0.5 g/L $CaCl_2 \cdot 2H_2O$, 0.5 g/L KCl, 3 g/L $MgCl_2 \cdot 6H_2O$, 22 g/L NaCl, 3 g/L Na_2SO_4, 1 mL trace elements (3 g $MgSO_4 \cdot 7H_2O$, 0.5 g $MnSO_4 \cdot H_2O$, 1 g NaCl, 0.1 g $FeSO_4 \cdot 7H_2O$, 0.1 g $CoCl_2 \cdot 6H_2O$, 0.1 g $CaCl_2$, 0.1 g $ZnSO_4 \cdot 7H_2O$, 0.01 g $CuSO_4 \cdot 5H_2O$, 0.01 g $AlK(SO_4)_2 \cdot 12H_2O$, 0.01 g H_3BO_3, and 0.01 g $NaMoO_4 \cdot 2H_2O$ in 1 L ddH_2O) and 1 mL vitamins (10 mg Pyridoxine hydrochloride, 5 mg thiamine-HCl, 5 mg riboflavin, 5 mg nicotinic acid, 5 mg D-calcium pantothenate, 5 mg P-Aminobenzoic acid, 5 mg thioctic acid, 2 mg biotin, 2 mg folic acid, and 0.1 mg vitamin B_{12} in 1 L ddH2O), supplemented with 3% (w/v)

of the corresponding sole carbon source (aspen lignocellulose, pine lignocellulose, and rice straw) and 50 μg/mL nystatin (catalog # N814558, Aladdin, Shanghai, China) [4, 5]. The lignocellulose substrates were collected from either tree plantation or rice field, as previously described [3, 6]. Each culture was incubated at 30℃, 150 rpm for 7 days, as our previous study suggested higher specific growth rate and lignocellulose degradation were observed at 30 ℃, compared to lower temperature (i.e., 15 ℃) [3]. Two negative controls, without either lignocellulose substrate or coastal sample inoculation, were set up under the same conditions. From Transfer 1 (T1) to Transfer 3 (T3), a 5 mL culture suspension at day 5 was successively transferred to 100 mL of fresh MB medium. Each enriched community (T1-T3) was collected at day 7 and stored in 1% (v/v) glycerol (final concentration) at -80℃. The culture experiments were performed with six biological replicates.

Quantitative PCR amplification (qPCR)

qPCR for the 16S rRNA gene was employed to monitor the growth of the bacterial communities. A 2 mL sample of culture was collected every day (from day 0 to 7) to extract genomic DNA by the CTAB method [6]. We used the 16S rRNA gene universal primer set 341F/519R (341F: 5′-CCTACGGGWGGCWGCA-3′ and 519R: 5′-TTACCGCGGCKGCTG-3′). qPCR was performed with a standard qPCR protocol [3] and quantified by a standard curve, generated from tenfold serially diluted DNA fragment of bacterial community 16S rRNA gene (10^{-1}-10^{-9} copy number/mL). Consequently, the cell abundance of each community was indicated by the 16S rRNA gene copy numbers/mL. Next, the specific growth rate (day^{-1}) was calculated as follows: $\mu = \frac{(\ln Nn - \ln Ni)}{N}$, where Ni/Nn is the initial/highest 16S rRNA gene copy number at the exponential growth phase, and N is the exponential growth days [7, 8]. The experiments were performed with six biological replicates.

Substrate measurement and analysis

The consumption of lignocellulosic substrate was measured by weighing method [9]. Briefly, the residual solid substrate in the culture was collected at day 7, filtered through nonwoven fabric (30 g/m^2, 30 × 30 cm), washed, dried, and then weighed. The culture without the inoculum was used as the blank control. The percentage of weight loss was used to indicate the lignocellulosic substrate consumed by the bacterial community.

The amounts of cellulose, hemicellulose, and lignin were examined by the Laboratory Analysis Protocol (LAP) of the National Renewable Energy Laboratory (NREL), Golden, CO, USA [10]. For lignin measurement, 0.5 g dried lignocellulosic sample was extracted by 95% (v/v) ethanol for 2 h using the Soxhlet method. 72% (w/w) H_2SO_4 was used to suspend the extracted slurry at 30 °C for 1 h, then was diluted to 4% (w/w) with milliQ pure water and incubated in an autoclave for 1 h at 121°C. It was subsequently filtered by filtering crucibles (25 mL, porcelain, medium porosity) and dried in Muffle furnace at 575°C for 4 h. The acid-soluble lignin content (ASL) was detected by UV-Vis spectroscopy at 320 nm, as previously reported [10]. The acid-insoluble lignin fraction was measured by the difference of weight loss [10]. For measurement of hemi-/cellulose compositions, a high-performance liquid chromatograph (HPLC, Agilent 1260 Infinity II) equipped with a refractive index detector (RID) was employed. An Agilent Hi-Plex H analysis column, with a flow rate of 0.4 mL/min 0.005 mol/L H_2SO_4, was used for HPLC analysis [11]. Glucose (catalog # G116303), xylose (catalog # X407961), and arabinose (catalog # L424561), were purchased from Aladdin, Shanghai, China, as the standard samples for the cellulose and hemicellulose hydrolysis products, respectively. They were also analyzed by HPLC using the same procedure [10]. The experiments were performed

with six biological replicates.

Soluble lignin derivates measurement

The soluble lignin derivates were analyzed by the gas chromatography-mass spectrometry (GCMS-QP2020 NX, Shimadu, Kyoto, Japan) [12]. Briefly, 3 mL of culture supernatant at day 5 from the ZA and DA consortia were collected by centrifugation (13000 g, 25℃, 8 min). Ethyl acetate (9 mL) was used to extract the supernatant and then 3 g anhydrous sodium sulfate was added to dewater. A 2 mL sample was collected through centrifugation (8000 g, 25℃, 10 min), dried by nitrogen gas stream, and mixed with 100 μL dioxane containing an internal standard (33 mg/mL ethylvanilline, catalog # E107635, Aladdin, Shanghai, China), 10 μL pyridine (catalog # P816290, Macklin, Shanghai, China), and 50 μL trimethylsilyl (BSTFA (N,O-bis (trimethylsilyl) trifluoroacetamide, catalog # 155195, Sigma-Aldrich, St. Louis, MO, USA) and TMCS (trimethylchlorosilane, catalog # 89595, Sigma-Aldrich, St. Louis, MO, USA) [13]. The solution was dissolved at 60℃ for 15 min, diluted 10-fold with hexane and injected into a GC-MS equipped with a HP-5MS column (thickness 0.25 μm; length 30 m; diameter 0.25 mm). The column temperature program was set as 50℃ hold for 5 min, 50–300℃ (10℃/min, hold time: 5 min) [12]. The compounds were identified by comparison with the GC-MS library database (NIST17). The relative abundance of each compound was calculated by the following formula, the abundance of each compound/ the abundance of ethylvanilline (the internal standard). The experiments were performed with three biological replicates.

Extracellular Enzyme Assays

Glucanase activity was measured via the Ghose method [14]. Specifically, 100 μL 1% (w/v) glucan solution (catalog # G684387, Aladdin, Shanghai, China) was mixed with 50 μL crude enzyme extract at 50 ℃ for 30 min. Then, 200 μL 1% (w/v) DNS

(3,5-Dinitrosalicylic acid, catalog # D109090, Aladdin, Shanghai, China) was added. Subsequently, the solution was boiled at 100 ℃ for 5 min. After cooling on ice, the OD540 value of the sample was measured to monitor the released glucose. One unit of enzyme activity was defined as the amount of enzyme that produced 1 μmol glucose per min [14].

Xylanase activity was measured via the DNS method [15]. 50 μL crude enzyme extract was mixed with 100 μL 1% (w/v) xylan (catalog # X140487, Aladdin, Shanghai, China) at 50 ℃ for 30 min. Then, 200 μL 1% (w/v) DNS solution was added and boiled at 100 ℃ for 5 min. The absorbance OD value of the sample was measured at 540 nm to monitor the released xylose. One unit of enzyme activity was defined as the amount of enzyme that generated 1 μmol xylose per min [15].

β-glucosidase activity was examined by the pNP-NPG method [16]. 50 μL of crude enzyme extract was added to 100 μL 5 mM p-NPG (p-Nitrophenyl-β-D-glucopyranoside, catalog # 487507, Sigma-Aldrich, St. Louis, MO, USA) in 0.05 M acetate buffer (pH = 4.5) and reacted at 50°C for 30 min. 1 mL 1 mol/L Na_2CO_3 was added immediately to terminate the reaction. The OD value at 405 nm was measured. One unit of enzyme activity was defined as the amount of enzyme that generated 1 μmol p-NP (p-Nitrophenol) per min [16].

β-xylosidase activity was measured by the pNP-NPX method [17]. 50 μL crude enzyme extract and 100 μL 5 mM p-NPX (4-Nitrophenyl-β-D-xylopyranoside, catalog # N130864, Aladdin, Shanghai, China) in 0.05 M acetate buffer (pH = 4.5) were mixed together and incubated at 50°C for 30 min. 1 mL 1 mol/L Na_2CO_3 was used to terminate the reaction. The OD value at 405 nm was measured. One unit of enzyme activity was defined as the amount of enzyme required to produce 1 μmol p-NP (p-Nitrophenol) per min [17].

High-throughput DNA sequencing

Three samples were randomly selected from each sampling site for 16S rRNA gene amplicon sequencing. Approximately 15 mL of culture sample was collected at day 5 for DNA extraction by the CTAB method [4], generating 200-1000 μg DNA per sample. 200 ng DNA per sample (n = 90) was used for 16S rRNA gene amplicon sequencing. The V4 region of the bacterial 16S rRNA gene, with unique barcode, was amplified using the primer set 515F/806R (515F: 5′-GTGCCA GCMGCCGCGGTAA-3′ and 806R: 5′-GGACTACHVGGG TWTCTAAT-3′). The amplified DNA fragments were sequenced using a NovaSeq 6000 System (Illumina) at Novogene Co., Ltd., Beijing, China, with 250 bp paired-end sequencing. An average of 105823 reads per sample were collected with a standard deviation of 9721.77. The sequencing data displayed high Good's coverage values (> 99%, Table S2) and have been deposited in the NCBI SRA database under the accession number PRJNA1029913.

Approximately 50 mL culture sample from ZA and DA consortia, with three biological replicates, was collected at day 5 for RNA extraction by the RNAprep Pure Cell/Bacteria Kit [18]. 500 ng RNA per sample (n = 6) were used for meta transcriptomics DNA sequencing using a NovaSeq 6000 System (Illumina) at Novogene Co., Ltd., Beijing, China, with 150 bp paired-end sequencing [19]. A total of 86168366 reads were collected, with an average of 14361394 reads per sample and a standard deviation of 4426112. The sequencing data have been deposited in the NCBI SRA database under the accession number PRJNA1029913.

High-throughput DNA sequencing data analysis

All 16S rRNA gene amplicon sequencing pair-end reads were analyzed using the DADA2 R package (version 1.3.2) and associated pipeline to remove the primer

sequences and chimeras, denoise, filter, and finally merge the sequences [20]. Sequencing results were subsequently rarefied to 63488 reads to normalize sequencing depth, based on the sample with the lowest sequence number. Consequently, amplicon sequence variants (ASVs) with 100% sequence identity [20] were generated and compared against the SILVA ribosomal RNA database (SILVA version 138.2), according to Silva alignment, classification, and tree service (Silva-ACT https://www.arb-silva.de/aligner/). Alignment was performed by the SINA aligner (version 1.2.12) with 70% identity cutoff [21].

For metatranscriptomic and metagenomic sequencing data, raw reads were firstly assessed and trimmed by Trimmomatic version 0.39 software [22] using the default parameters (LEADING:3 TRAILING:3 SLIDINGWINDOW:4:15 MINLEN:50). The quality-controlled reads of metatranscriptomic data were assembled by *de novo* assembly in Trinity version 2.8.5 software [23]. The quality-controlled reads of metagenomic data were co-assembled to contigs using MEGAHIT (version 1.2.9) [24] with the parameters (–k-list 21,29,39,59,79,99,119,141). The open reading frames (ORFs) for co-assembled metatranscriptomic and metagenomic sequencing reads were predicted by Prodigal (version 2.6.3) [25] in meta mode.

The carbohydrate-active enzymes (CAZy) database (http://www.cazy.org; CAZy update, April 2023) [26] and lignin catabolism database (LCdb) [27] were used for the lignocellulose degrading functional profiling analysis for the ORFs, with the DIAMOND program (-k 1 -e 0.0001 -id 0.3) [28]. The sequences of gene families were extracted using the Seqtk program (version 1.3) (https://github.com/lh3/seqtk) and analyzed by Salmon version 1.6.0 [29] to calculate the gene expression levels for metatranscriptomic sequencing data, with default parameters (-k 31). CD-HIT (version 4.8.1) was used to de-replicated for ORFs from metagenomic assemblies [24]

at 95% sequence identity and 80% alignment coverage (-c 0.95 -aS 0.8). Bowtie2 (version 2.4.5) was employed for the index construction and generation of gene-centric read mapping [30]. Sorted BAM files, which was generated by SAMtools (version 1.3.1) [30], were employed to quantify the relative abundance of genes using CoverM (version 0.6.1) [31] in contig mode. Taxonomic assignment was performed by the Kraken2 tool (version 2.1.3), as previously described [32, 33].

For the association analysis between metabolic specialist/generalist and habitat specialist/generalist, the taxonomic information at the species level were extracted from metatranscriptomic sequencing data, by the Kraken2 tool (version 2.1.3). The corresponding 16S rRNA gene sequences were downloaded via the EzBioCloud Database (https://www.ezbiocloud.net) and then blasted against ASV sequences in this study with 100% identity [34].

Consortium diversity and variance analysis

Shannon index was calculated to characterize alpha diversity of cultured community at the levels of taxonomy and functional genes using the previously reported method [35]. Non-metric multidimensional scaling (NMDS) plot was performed based on Bray–Curtis dissimilarities to evaluate differences in beta diversity [36]. Redundancy analysis (RDA) was conducted to identify the contribution of factors (temperature, latitude, and substrate) to the beta diversity of the cultured communities [37]. To further quantify the effects of the environmental factors, PERMANOVA was performed through the adonis2 function in the vegan package, based on Bray–Curtis dissimilarities among Hellinger-transformed counts and 999 permutations [38]. To investigate the relationship between temperature/latitude and the community diversity/activity (Shannon index, growth rate, and substrate degradation), linear regression analysis was carried out based on Spearman's

correlation coefficients. They were performed using R (version 4.2.1) package ‘psych’ (version 2.2.9). Procrustes analysis was performed to determine association between taxonomic and functional gene compositions of ZA and DA bacterial consortia based on NMDS [39], using R (version 4.2.1) and R package ‘vegan’ (version 2.4.6).

Null model

Null model analysis (iCAMP), with 16S rRNA gene amplicon sequencing data, was performed to evaluate the relative contributions of deterministic and stochastic processes, as previously described [32, 40]. Briefly, a total of 1000 null models were firstly generated via each calculated Bray–Curtis dissimilarity. An average Bray–Curtis dissimilarity matrix was subsequently generated. Community assembly stochasticity was calculated through comparing the observed and randomized community dissimilarity. The normalized stochasticity ratio (NST), with the threshold at 50% was used to quantify the relative contributions for the community assembly. NST < 50% indicates more deterministic assembly. The NST package (version 3.1.10) in R (version 4.2.1) was used for this analysis [40].

Fittings of MTE

Shannon index, specific growth rate, lignin degradation, and lignocellulose degradation were used to test the hypothesis that cultured community diversity and metabolism follow the exponential effects of environmental temperature. In the linear model of MTE, $\ln(\mathrm{R}) = a - E_a \times \frac{1}{KT}$, K is Boltzmann’s constant (8.6173324×10^{-5}), T is absolute annual average temperature in Kelvin (K) [41] and a is the intercept of this linear model [42]. E_a represents the activation energy, which is the inverse number of the slope in the linear regression [42]. R is the above-mentioned community parameters.

Statistical analysis

All statistical analyses were performed by R studio software (version 4.2.1). For comparison of the beta diversity between two taxonomic groups enriched on different substrates or from high/low latitudes (Table S3), the complementary nonparametric statistical analyses and the statistical significance of ANOSIM, Adonis, MRPP, and response ratio analyses were performed in R studio software (version 4.2.1) with the vegan (version 2.6.4) package [43, 44]. The Bray–Curtis dissimilarities were used to calculate the dissimilarities among consortia [45]. Through the Kruskal-Wallis test, indicator species analysis (IndVal) and linear discriminant analysis (LDA) effect size (LEfSe) were employed to identify differentially abundant ASVs between high and low latitudinal groups that showed a spatial separation, based on Bray–Curtis dissimilarities. The LDA score threshold was set to 3.0. The ‘indicspecies’ package for IndVal and ‘microeco’ package for LEfSe was used [46, 47]. R studio software (version 4.2.1) was employed for the Kruskal-Wallis test [48]. Statistical differences between two groups were compared by two-tailed Student's *t*-test [49], where $P < 0.05$ was considered statistically significant, and $P < 0.01$ was highly significant.

Text S2 Results

Compositions of communities that were enriched on different substrates

Aspen consortia were rich in the genera *Paenibacillus* (20.1%), *Salinimicrobium* (6.1%), and *Gramella* (5.3%), whereas pine consortia contained abundant *Vibrio* (9.5%), *Paenibacillus* (6.7%), and *Pseudomonas* (6.1%). In contrast, rice straw recruited the genera with high abundance (e.g., *Bacillus* (18.2%), *Paenibacillus* (18%), and *Heyndrickxia* (6.7%)) (Fig. S7).

Compositions of communities that were cultured from high and low latitudes

30.4% of amplicon sequence variants (ASVs) showed significantly higher abundances in the high latitude group (P < 0.05, Fig. 3D). They recruited more members of the genus *Paenibacillus*, including ASV18, ASV24, ASV29, and ASV31, of which *Paenibacillus* ASV29 was observed only in high latitudes. In addition, *Oceanisphaera* (ASV22 and ASV134), *Lactobacillus* ASV28, and *Peribacillus* ASV15 were more abundant in high latitudes, indicating that they are habitat specialists. 14.8% of ASVs showed significantly higher abundance in the low latitude group, including *Bacillus* ASV5, *Owenweeksia* ASV19, *Idiomarina* ASV21, and *Pseudohoeflea* ASV23 (Fig. 3D and Fig. S8). Specifically, a significant latitude decay pattern was observed for the genus *Bacillus* (ASV5, ASV306, ASV307, and ASV374, Fig. S9). Moreover, we observed habitat generalists that were cultured across the sampled coastal zones, including *Paenibacillus* (ASV4, ASV8, and ASV13), *Bacillus* ASV2, *Priestia* ASV9, *Vibro* ASV3, *Heyndrickxia* ASV7, and *Gracilobacillus* ASV12 (Fig. 3D and Fig. S8).

Similar expression levels of genes involved in hemi-/cellulose hydrolysis

For cellulose hydrolysis, the expression of the GH2, GH3, GH5, GH11, GH16, and GH55 gene families were identified in both sets of consortia (Fig. S10A-B and Table

S5b). These gene families encode glucanases and glucosidases, which hydrolyze cellulose to generate glucose [50] . Expression of the GH3, GH5, GH30, GH11, GH43, and GH52 gene families, which encode xylanases and xylosidases for hemicellulose hydrolysis, generating xylose [51], were also identified in the ZA and DA consortia (Fig. S10A-B and Table S5b). In addition, the expression of the AA3, AA5, AA6, and AA10 gene families which encode auxiliary enzymes to assist hemi-/cellulose hydrolysis [52], were also identified (Fig. S10A-B). The expression levels were mostly similar between the ZA and DA consortia without significant changes ($P > 0.05$), the exceptions being GH3, GH8, GH16, GH55, and AA10 (Fig. S10A-B). Furthermore, the corresponding enzyme activities were very similar (1.18-fold change, Fig. S10C). Overall, a similar pattern was observed for hemi-/cellulose degradation (1.15-fold change, Fig. S10D-E), without substantial biological differences between ZA and DA consortia.

Lignin depolymerization enzyme groups of ZA and DA

The *poxB* encodes pyruvate oxidase, which provides H_2O_2 as the electron acceptor for DypB mediated lignin oxidation [53, 54]. The *nuoE* gene family encodes NADH-quinone oxidoreductase, which participates in quinone redox cycling to metabolize lignin depolymerization products [55]. The *gox* gene family encodes glycolate oxidase, which alleviates the accumulation of aldehyde during lignin oxidation [56]. These auxiliary enzymes assist *dypB* during lignin depolymerization in ZA.

The DA consortia with their higher expression level of *dypA*, also highly expressed *cat* and *gpx*, which encode catalase and glutathione peroxidase, respectively (Fig. 5D and Fig. S11A-B). These latter two enzymes participate in reactive oxygen species (ROS) cycling and here, as auxiliary enzymes, assist *dypA* in lignin depolymerization.

Supplementary Figures

Figure S1 Growth (A), cellulose degradation (B), and hemicellulose degradation (C) of the cultured costal bacterial lignocellulose degrading communities. Three lignocellulosic substrates, aspen, pine, and rice straw, were used, respectively. The maximal 16S rRNA gene copy number at day 5 was used to indicate community growth at T3. Data are presented as mean values ± standard deviation, n = 6 biological replicates.

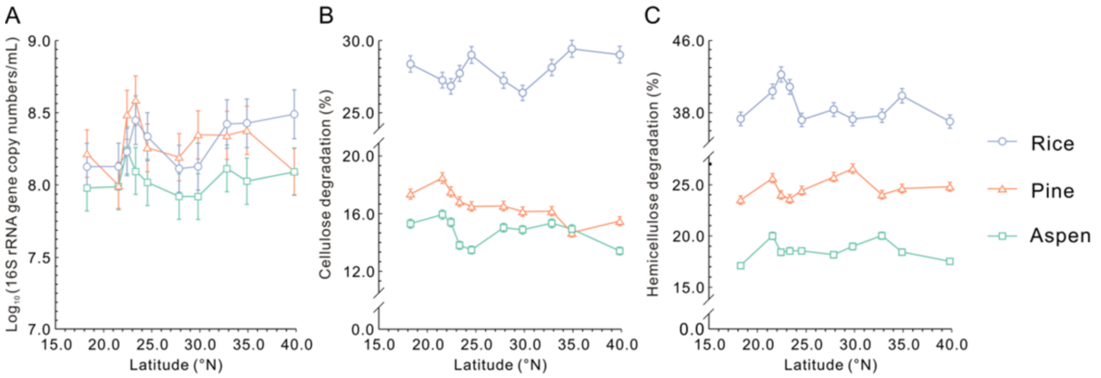

Figure S2 Relative abundances of lignocellulose degrading functional traits along the coastline of China, spanning from the southmost (SanYa) to the northmost (DanDong) (Supplementary Table S1). Data are from eight biological replicates.

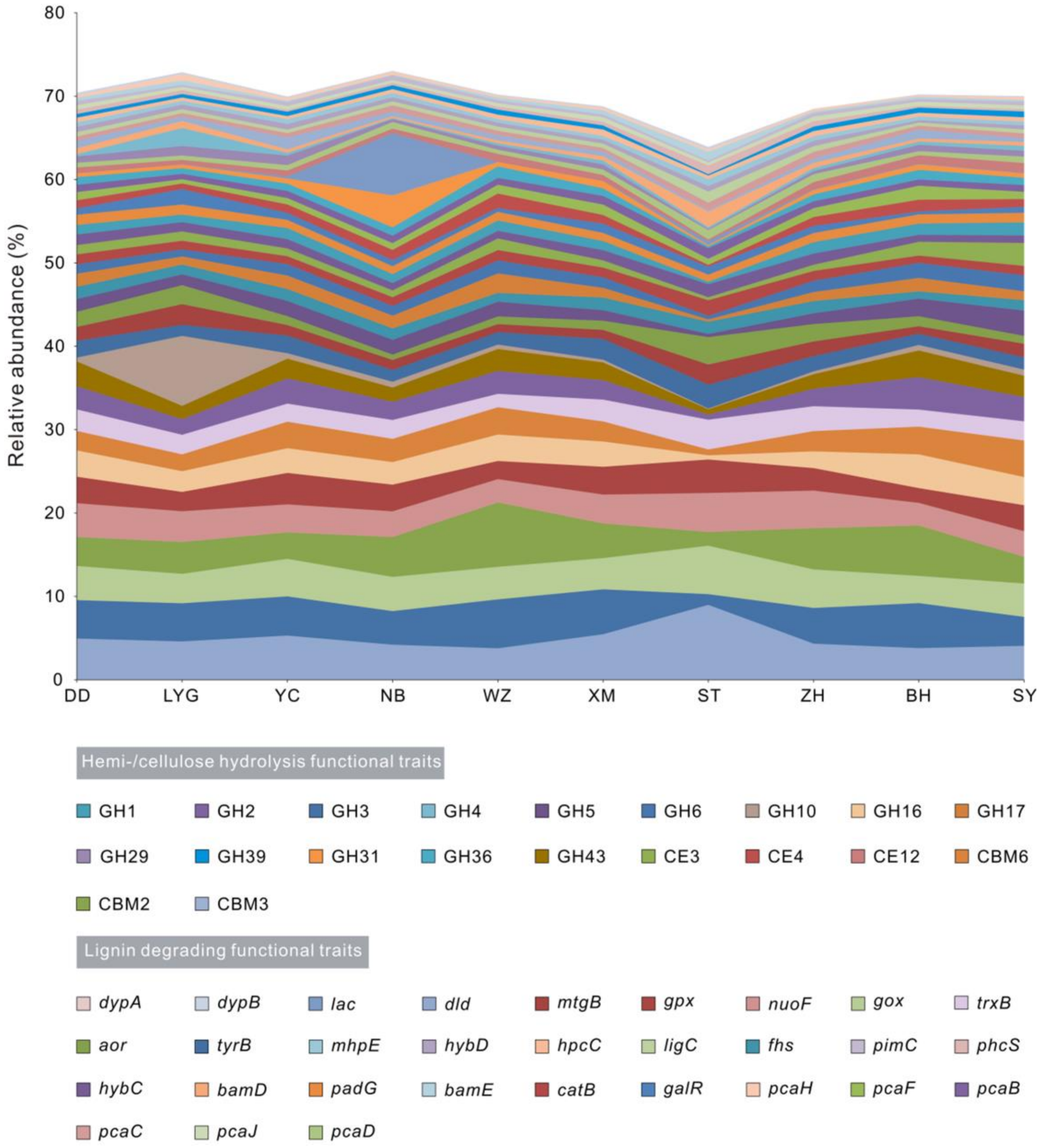

Figure S3 Relationships between community metabolism and environmental temperature. (A-C) Scatterplots of environmental temperature vs. specific growth rate (A), lignocellulose degradation (B), and lignin degradation (C). (D-I) Scatterplots of temperature vs. specific growth rate (D and G), lignocellulose degradation (E and H), and lignin degradation (F and I) of pine and rice degrading consortia. The natural log of specific growth rate, lignocellulose degradation, and lignin degradation values were used for analyzing the relationships between community metabolism and temperature, which is presented as the inverse of annual average temperature in degrees Kelvin. Each line represents the least squares regression fit and the shaded area represents the 95% confidence interval. Dashed line indicates a non-significant relationship. n = 6 biological replicates.

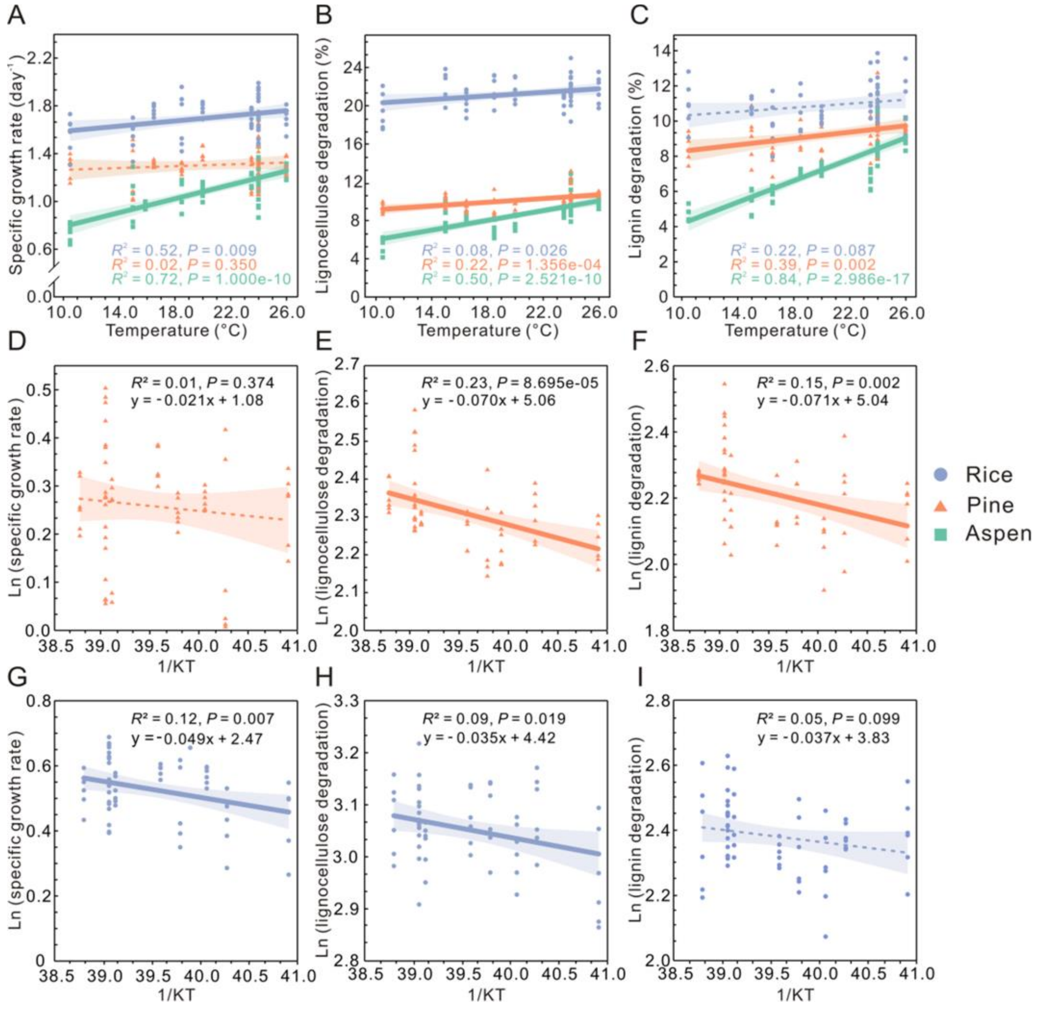

Figure S4 Relationships between community diversity and environmental variables. (A) The scatterplot of Shannon index vs. latitude. (B-D) The relationship between community alpha diversity and environmental temperature for *in situ* lignocellulose degrading (B) and cultured consortia enriched on pine (C) and rice (D) substrate. The natural log of Shannon index values was used for analyzing the relationships between community diversity and temperature, which was expressed as the inverse of annual average temperature in degrees Kelvin. Each line represents the least squares regression fit and the shaded area represents the 95% confidence interval. Dashed line indicates a non-significant relationship. (E) Redundancy analysis (RDA) of consortia based on the Bray-Curtis dissimilarities. Data are from three biological replicates for cultured consortia and six biological replicates for *in situ* consortia.

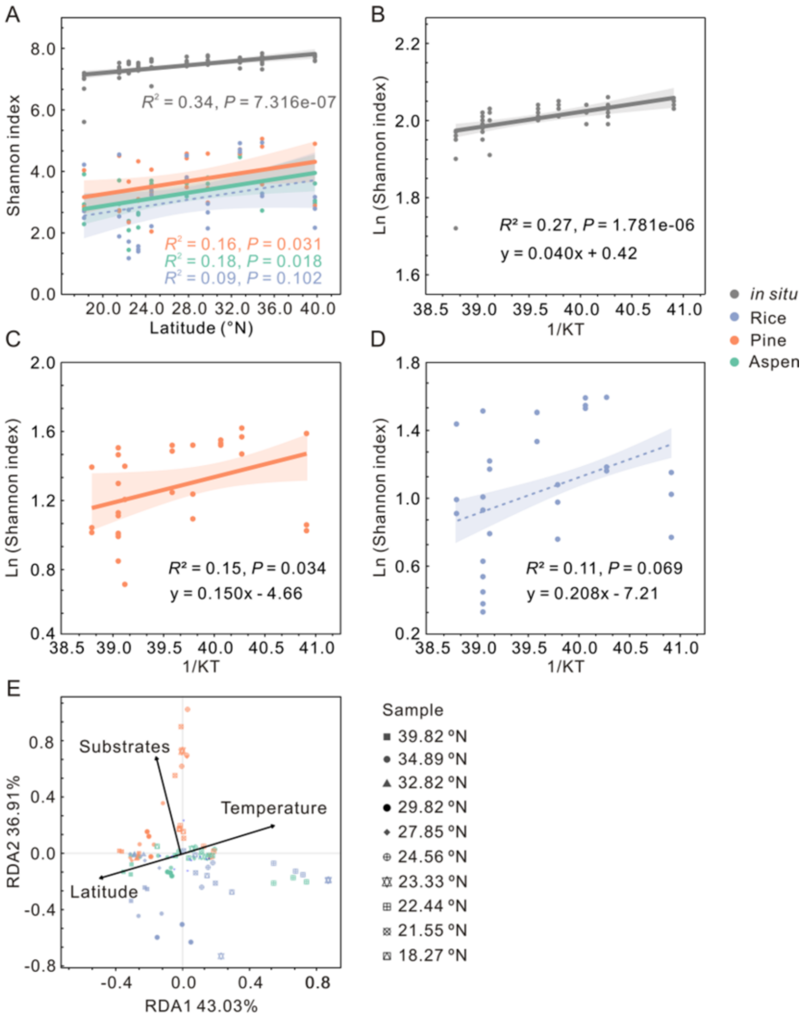

Figure S5 Relationships between temperature and community diversity of aspen degraders. Scatterplots of temperature vs. aspen community diversity of taxa that expressed lignocellulose degrading genes (A and C) and lignin degrading genes (B and D). The natural log of diversity for aspen lignocellulose degraders and aspen lignin degraders, as indicated by Shannon index, were used for analyzing the relationships between aspen community diversity and temperature, which was expressed as the inverse of the annual average temperature in degrees Kelvin. Each line represents a least squares regression fit and the shaded area represents the 95% confidence interval, n = 3 biological replicates.

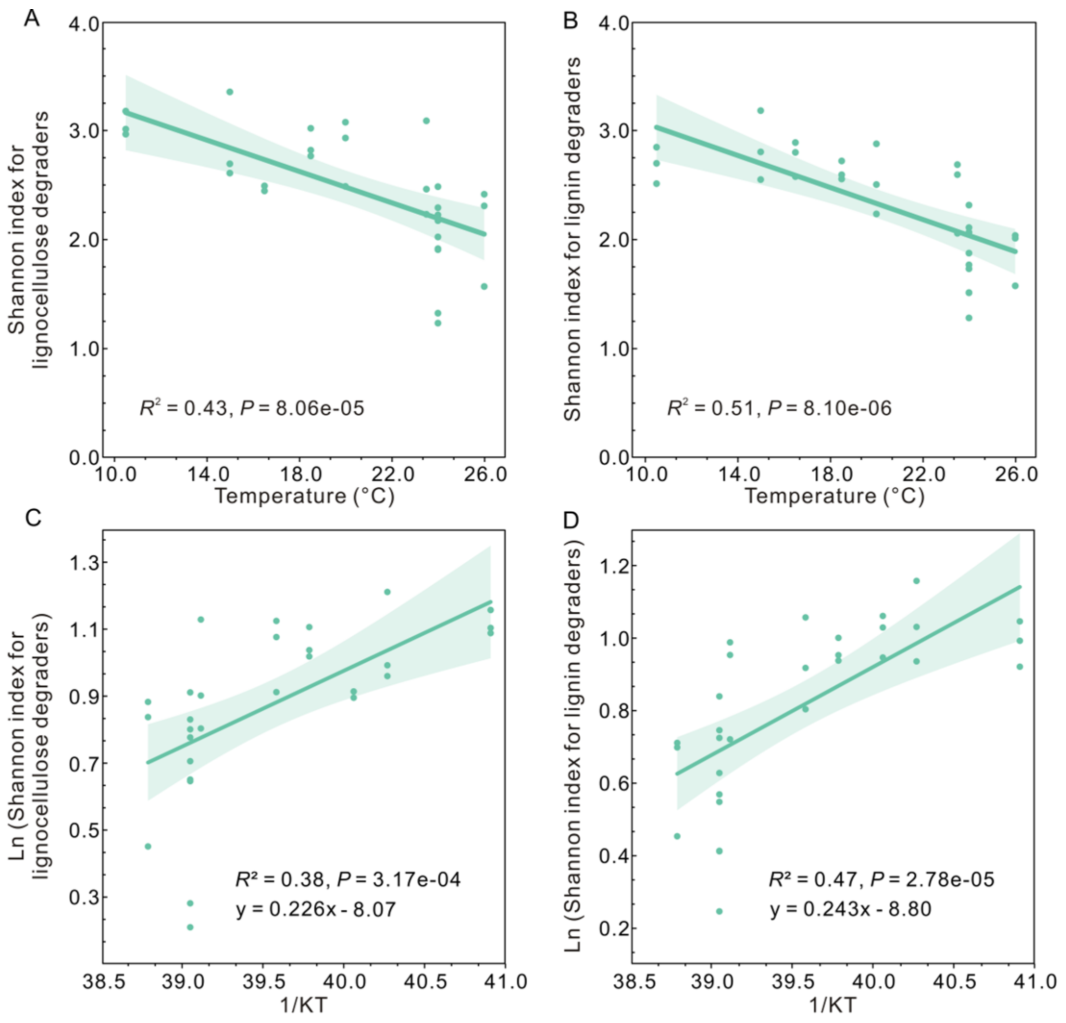

Figure S6 Assembly of the culturable bacterial consortia from Chinese coasts. Stochastic ratio represents the stochasticity of community assembly for taxonomic groups from different latitudes (A) and enriched on different substrates (B).

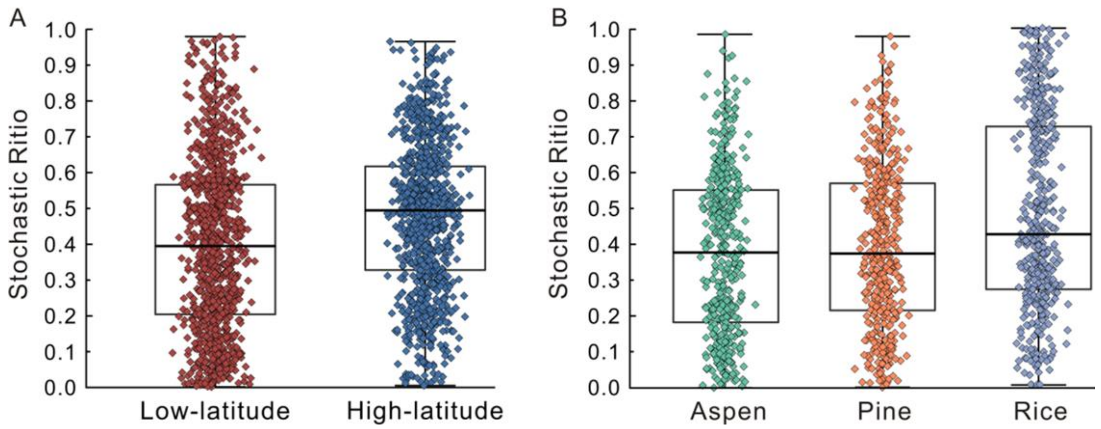

Figure S7 Compositions of the cultured bacterial consortia along the Chinese coasts, enriched on different substrates. Only bacterial genera with relative abundance > 0.1% are shown. Data are presented as mean values, n = 3 biological replicates. A: aspen, P: pine, R: rice.

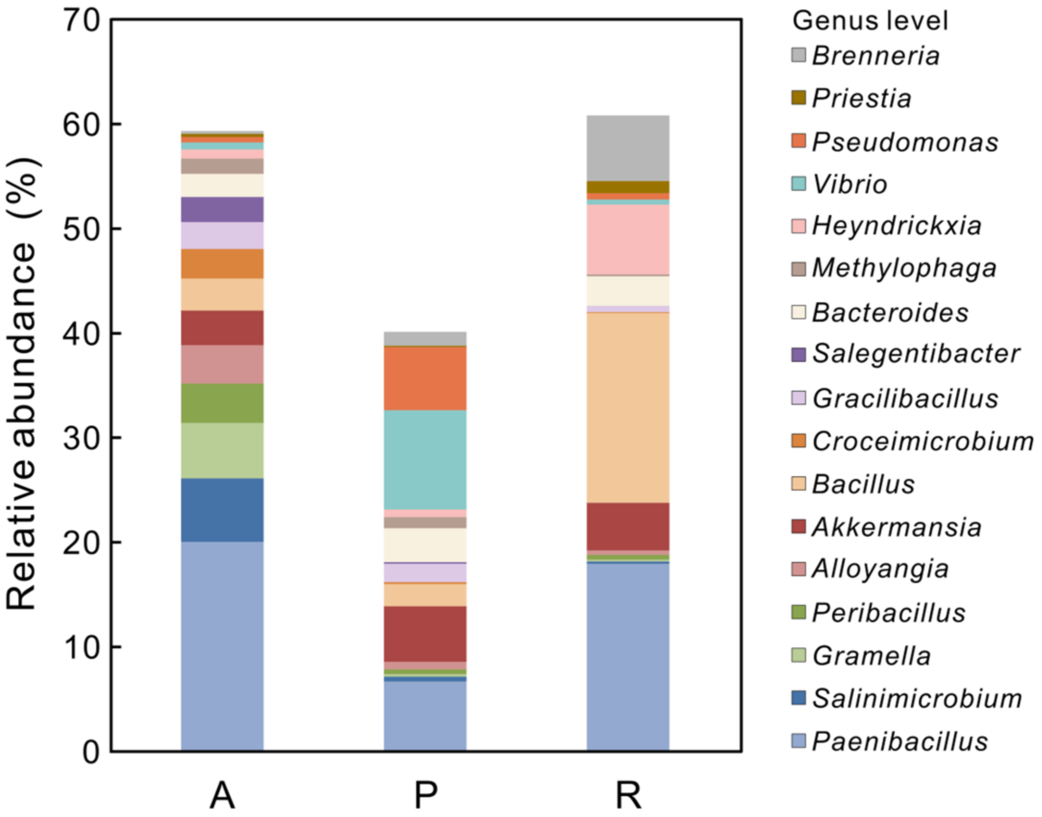

Figure S8 Distribution of ASVs along the Chinese coasts. Heat map displays log_2-transformed relative abundance of ASVs (> 0.5%). Hierarchical clustering of ASVs is based on Ward’s method. Generalists: ASVs that are widespread along the Chinese coasts, without significant difference ($P > 0.05$). Specialists: ASVs that show significantly higher abundance ($P < 0.05$) in either high latitude (HL) or low latitude (LL). Data are presented as mean values, n = 3 biological replicates.

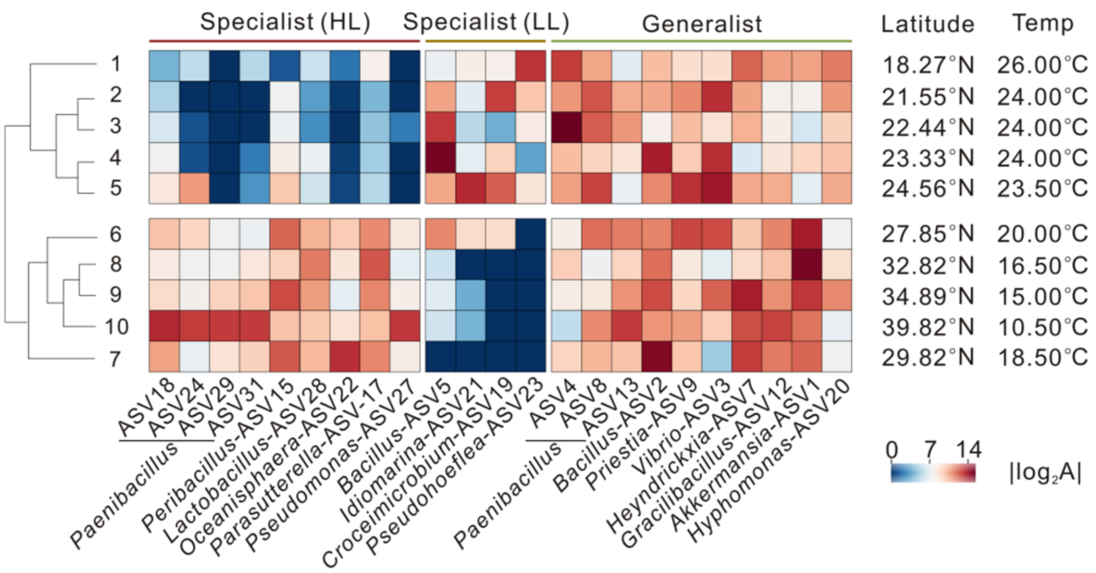

Figure S9 Latitude decay pattern for *Bacillus* ASVs. The line represents a least squares regression fit and the shaded area represents the 95% confidence interval. n = 3 biological replicates.

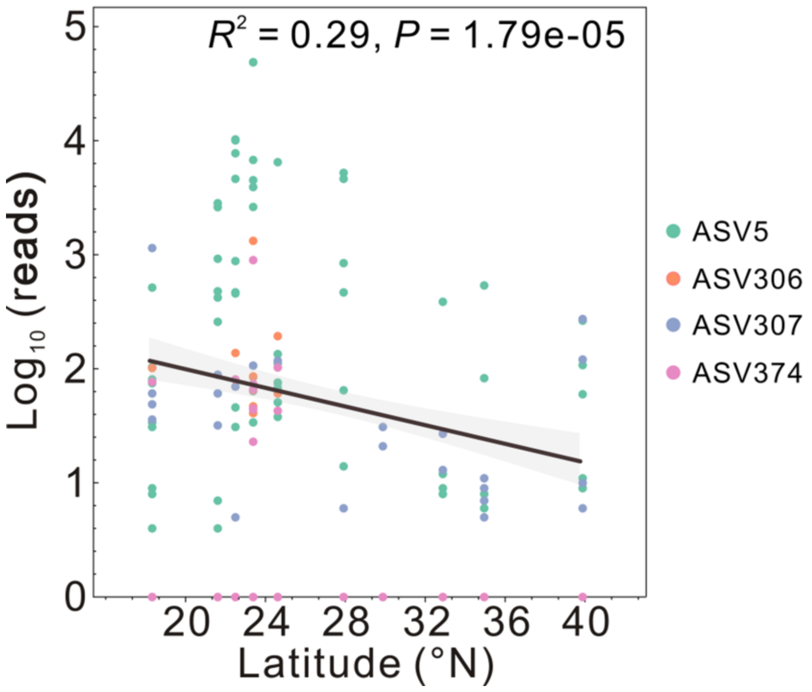

Figure S10 Comparisons of the hemi-/cellulose degradation between ZA and DA consortia. (A) The pathways for hemi-/cellulose hydrolysis, which were used by ZA and DA consortia. (B) The expression levels of relevant functional genes involved in hemi-/cellulose hydrolysis. Heatmap displays the "$\log_2$" normalized relative expression values (each gene reads/the total lignocellulose degrading gene reads). n = 3 biological replicates. (C) The enzyme activities for hemi-/cellulose hydrolysis. n = 3 biological replicates. (D) HPLC analysis for hexose and pentose generated during hemi-/cellulose hydrolysis. (E) Hemi-/cellulose degradation by ZA and DA culturable consortia, n = 6 biological replicates. The significance of differences was evaluated by two-sided Student's t-test (B–E): * P < 0.05, ** P < 0.01. Data are presented as mean values, the error bars (C and E) represent mean values ± standard deviation.

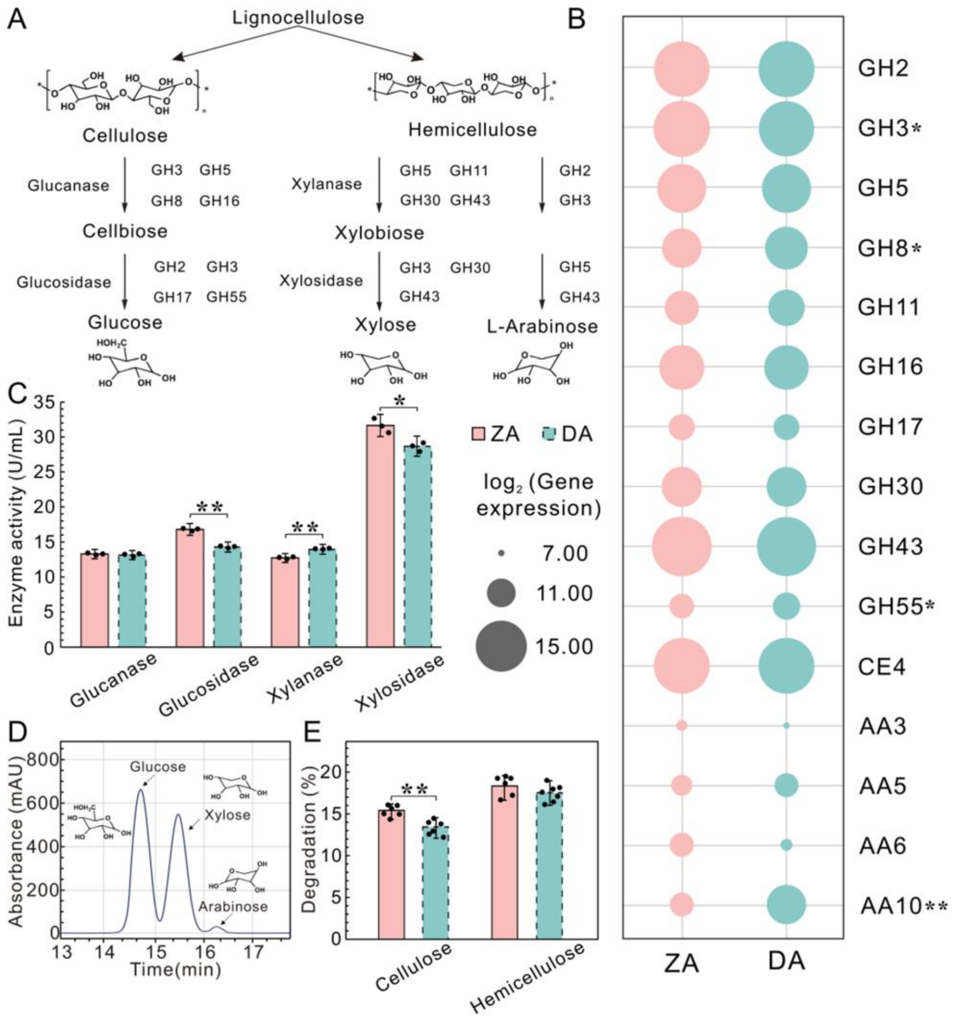

Figure S11 Comparison of the lignin degrading gene expression levels between ZA and DA consortia. (A) The expression levels of relevant functional genes involved in lignin depolymerization. (B) Proportional representation of specifically expressed lignin depolymerization gene families. (C) The expression levels of gene families involved in lignin-derived aromatic compound degradation. Heatmap displays the "$\log_2$" normalized relative expression values (each gene reads/the total lignocellulose degrading gene reads). White grid indicates that the gene was not expressed. *: $P < 0.05$, **: $P < 0.01$, ***: $P < 0.001$. (D) Proportional representation of specifically expressed gene families involved in degradation of G-, H-, and S-type lignin units. (E) Proportion of pathways with statically significant differences among the total pathways of each type lignin unit in ZA and DA consortia. Data (A-E) are from three biological replicates.

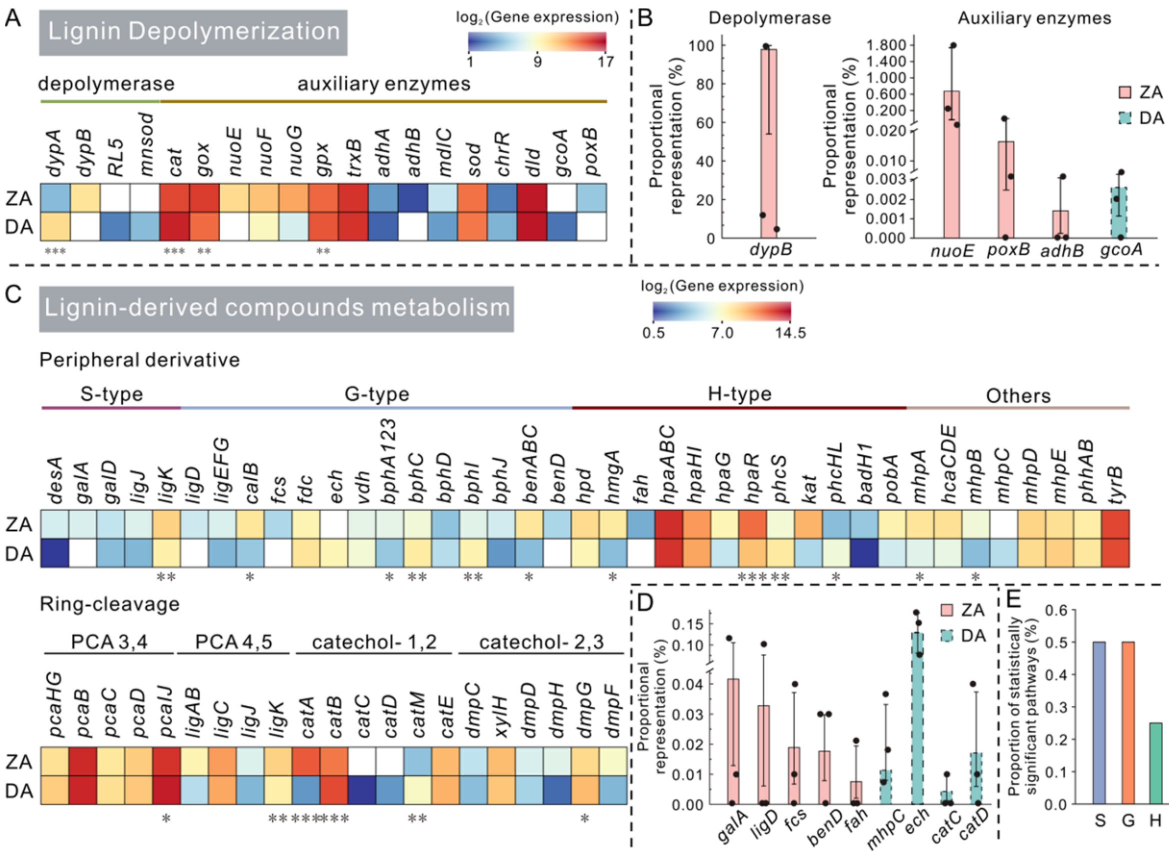

Figure S12 Bacterial compositions of the cultured and *in situ* lignocellulose degrading consortia at Zhuhai (ZH) and Dandong (DD). Species with over 0.3% relative abundance in ZA (A) and DA (B) are shown. Data are from three biological replicates for ZA and DA consortia and eight biological replicates for *in situ* consortia.

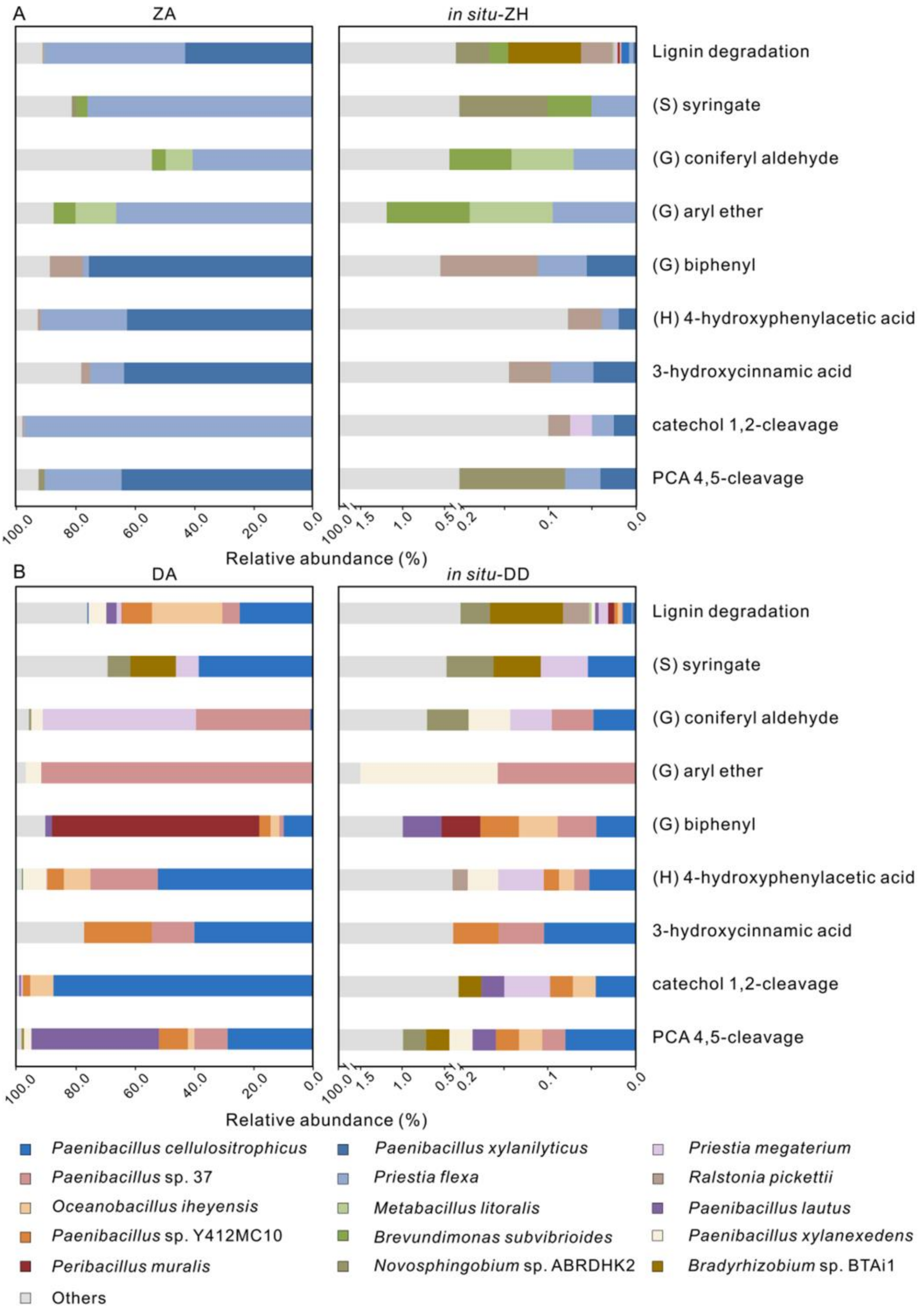

Figure S13 Relative abundances of the taxonomic and functional groups in *in situ* and cultured lignocellulose degrading consortia. (A) The relative abundance of the species in *in situ* lignocellulose degrading consortia (left) and cultured consortia (right). Species with over 0.3% relative abundance in *in situ* lignocellulose degrading consortia are shown. (B) The relative abundance of the functional groups in *in situ* lignocellulose degrading consortia and cultured consortia. Data are from three biological replicates for cultured consortia and eight biological replicates for *in situ* consortia. 4-HP: 4-hydroxyphenylacetic acid.

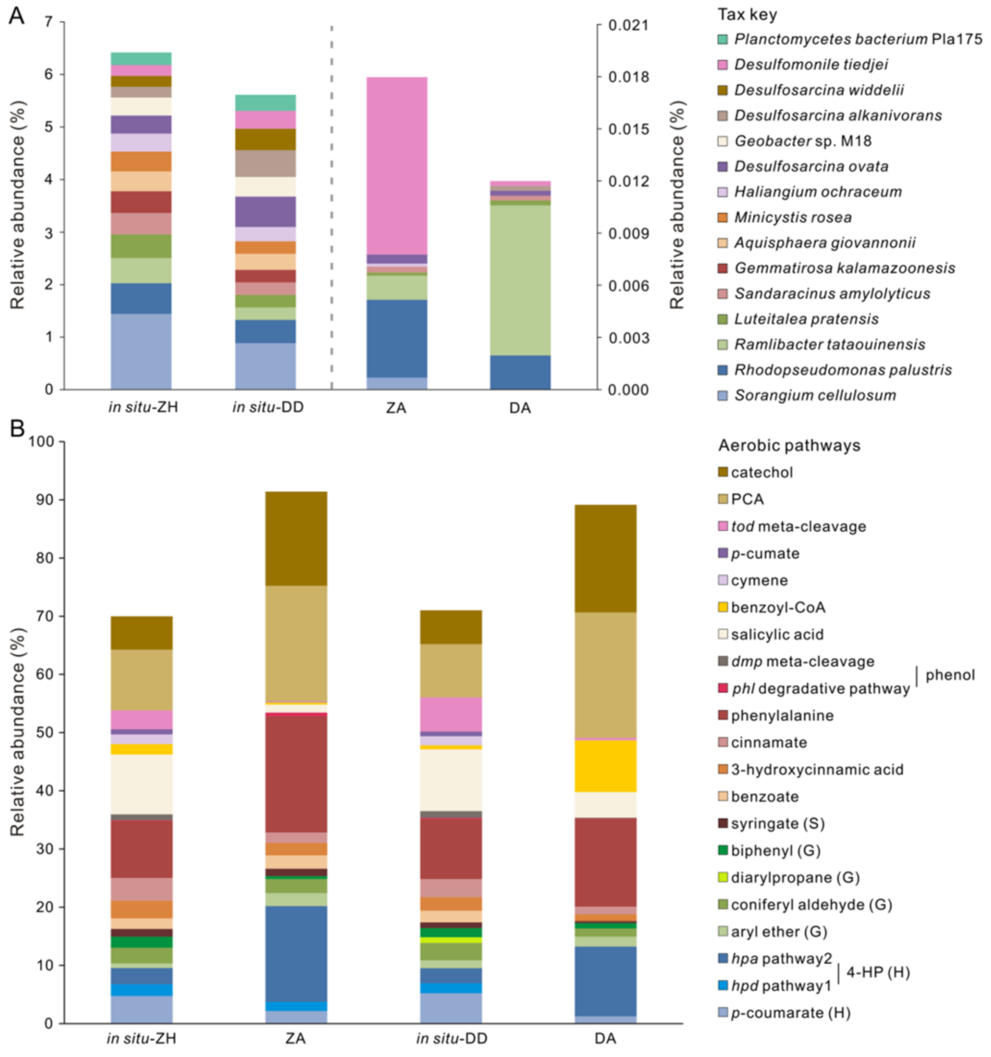

Figure S14 Procrustes analysis of taxonomy and functional gene compositions in ZA and DA consortia. Sample groups are color-coded. Line segments link 16S rRNA gene amplicon and metatranscriptome sequencing data of the ZA and DA consortia, based on NMDS.

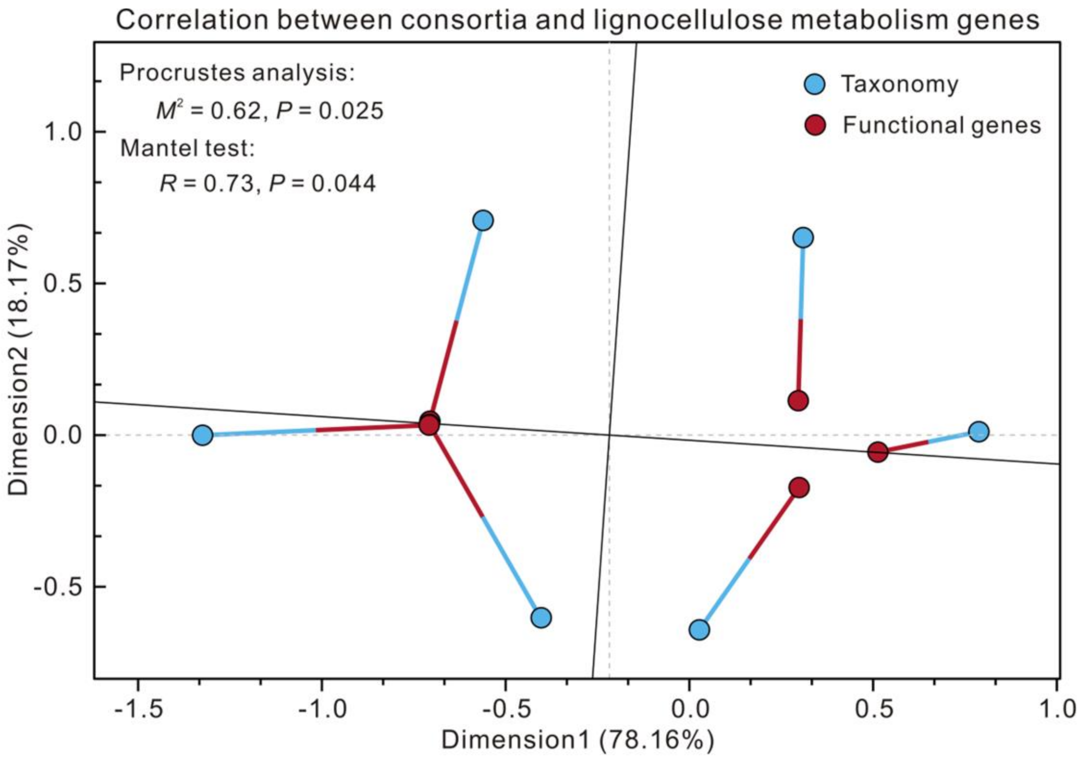

Figure S15 Abundances of metabolic specialists/generalists along annual average temperature in Chinese coasts. Each line represents the least squares regression fit and the shaded area represents the 95% confidence interval. Dashed line indicates a non-significant relationship. n = 3 biological replicates.

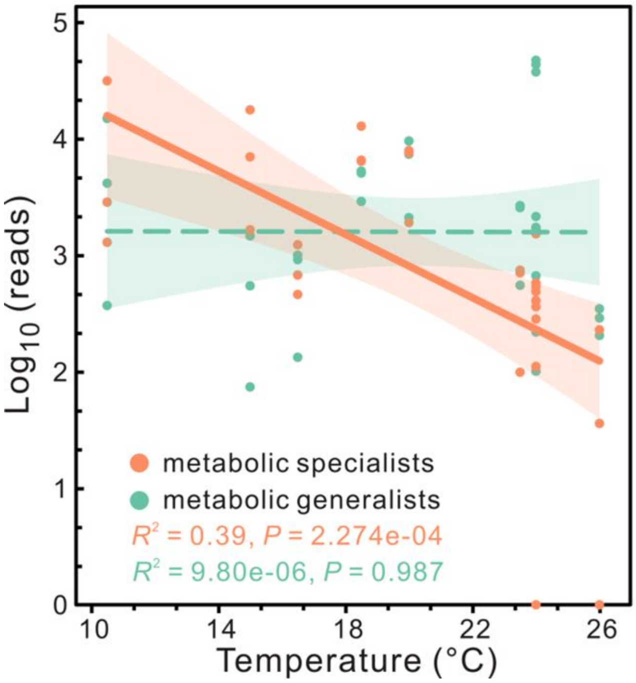

Supplementary Tables

Table S1 Geographic information for the ten sampling sites along the Chinese coast. The temperature data were downloaded from the website (https://www.tianqi24.com/). The additional data were downloaded from WorldClim2 (https://www.worlpdclim.org/).

Table S2 Richness, Shannon, Simpson, Pielou, and Good's Coverage's index for the 16S rRNA gene amplicon sequencing data. n = 3 biological replicates.

Notes: D: Dandong. L: Lianyungang. Y: Yancheng. N: Ningbo. W: Wenzhou. X: Xiamen. S: Shantou. Z: Zhuhai. B: Beihai. SanA: Sanya. A: aspen. P: pine. R: rice.

Table S3 Significance tests of beta diversity differences among the communities.

Communities	PERMANOVA		ANOSIM		MRPP	
	F	*P*	*R*	*P*	δ	*P*
Aspen versus Rice	2.51	0.003	0.10	0.005	0.02	0.007
Aspen versus Pine	2.58	0.005	0.10	0.006	0.02	0.002
Rice versus Pine	3.28	0.001	0.19	0.001	0.02	0.003
High latitude versus Low latitude	3.56	0.002	0.08	0.001	0.02	0.002

Table S4 Lignocellulosic composition and functional group contents of lignin in different lignocellulose sources.

Substrate	Composition (%)			Relative molar composition of the lignin aromatic units (%)		
	Cellulose	Hemicellulose	Lignin	*p*-hydroxyphenyl OH	Guaiacyl OH	Syringyl OH
Aspen	44.9	28.7	25.5	32.8	41.0	26.2
Pine	43.6	21.2	24.2	17.4	82.6	0.0
Rice	36.1	26.4	15.8	44.2	55.8	0.0

The data were collected from [6, 57].

Table S5 Relative expression levels of lignin degrading gene families (a) and hemi-/cellulose degrading gene families (b) in ZA and DA consortia.

Table S6 Lignocellulose degradation by cultured intertidal bacterial consortia with/without pervious *in situ* enrichment.

Substrate		Latitudes (°N)	*in situ* enrichment	Degradation (%)	*P* value
Aspen	lignin	29.95	+	8.6 ± 1.7	\
		27.85	-	7.2 ± 0.3	0.08
		29.82	-	7.1 ± 0.1	0.05
	cellulose	29.95	+	19.3 ± 0.6	\
		27.85	-	15.1 ± 0.3	0.00
		29.82	-	14.9 ± 1.9	0.00
	hemicellulose	29.95	+	22.4 ± 0.3	\
		27.85	-	18.2 ± 1.2	0.00
		29.82	-	19.2 ± 1.0	0.00
Pine	lignin	29.95	+	9.8 ± 1.3	\
		27.85	-	8.6 ± 0.7	0.07
		29.82	-	9.1 ± 0.7	0.23
	cellulose	29.95	+	13.3 ± 0.8	\
		27.85	-	16.6 ± 0.8	0.00
		29.82	-	16.2 ± 1.3	0.00
	hemicellulose	29.95	+	27.4 ± 3.4	\
		27.85	-	26.0 ± 1.5	0.39
		29.82	-	26.9 ± 1.8	0.79

		29.95	+	8.1 ±2.1	\
	lignin	27.85	-	10.3 ±0.4	0.03
		29.82	-	10.4 ±1.2	0.05
		29.95	+	28.7 ±2.4	\
Rice	cellulose	27.85	-	27.3 ±1.4	0.23
		29.82	-	26.4 ±0.9	0.05
		29.95	+	36.4 ±2.8	\
	hemicellulose	27.85	-	38.3 ±0.8	0.13
		29.82	-	37.3 ±1.8	0.52

Notes: "+/-": with/without 6-month *in situ* lignocellulose enrichment.

P value for substrate degradation between consortia with and without previous *in situ* enrichment.

This is in comparison with our previous study [3].

Reference

1. Ji M et al. Biodiversity of mudflat intertidal viromes along the Chinese coasts. *Nat Commun*. 2024;**15**:8611 https://doi.org/10.1038/s41467-024-52996-x
2. Ma K et al. Disentangling drivers of mudflat intertidal DOM chemodiversity using ecological models. *Nat Commun*. 2024;**15**:6620 https://doi.org/10.1038/s41467-024-50841-9
3. Ma W, Lin L, Peng Q. Origin, selection, and succession of coastal Intertidal zone-derived bacterial communities associated with the degradation of various lignocellulose substrates. *Microb Ecol*. 2023;**86**:1589-603 https://doi.org/10.1007/s00248-023-02170-5
4. Wang X, Lin L, Zhou J. Links among extracellular enzymes, lignin degradation and cell growth establish the models to identify marine lignin-utilizing bacteria. *Environ Microbiol*. 2021;**23**:160-73 https://doi.org/10.1111/1462-2920.15289
5. Peng Q et al. Modeling bacterial interactions uncovers the importance of outliers in the coastal lignin-degrading consortium. *Nat Commun*. 2025;**16**:639 https://doi.org/10.1038/s41467-025-56012-8
6. Peng Q et al. Unraveling the roles of coastal bacterial consortia in degradation of various lignocellulosic substrates. *mSystems*. 2023;**8**:e0128322 https://doi.org/10.1128/msystems.01283-22
7. Cortes-Tolalpa L et al. Halotolerant microbial consortia able to degrade highly recalcitrant plant biomass substrate. *Appl Microbiol Biotechnol*. 2018;**102**:2913-27 https://doi.org/10.1007/s00253-017-8714-6
8. Carroll J, Van Oostende N, Ward BB. Evaluation of genomic sequence-based growth rate methods for synchronized *Synechococcus* cultures. *Appl Environ Microbiol*. 2022;**88**:e0174321 https://doi.org/10.1128/aem.01743-21
9. De Lima Brossi MJ et al. Soil-derived microbial consortia enriched with different plant biomass reveal distinct players acting in lignocellulose degradation. *Microb Ecol*. 2016;**71**:616-27 https://doi.org/10.1007/s00248-015-0683-7
10. Decker SR et al. High-throughput screening of recalcitrance variations in lignocellulosic biomass: total lignin, lignin monomers, and enzymatic sugar release. *J Vis Exp*. 2015;**103**:53163 https://doi.org/10.3791/53163
11. Kłosowski G, Mikulski D. Impact of lignocellulose pretreatment by-products on *S. cerevisiae* strain ethanol red metabolism during aerobic and an-aerobic growth. *Molecules*. 2021;**26**:806 https://doi.org/10.3390/molecules26040806
12. Cao L et al. Efficient extracellular laccase secretion via bio-designed secretory apparatuses to enhance bacterial utilization of recalcitrant lignin. *Green Chem*. 2021;**23**:2079-94 https://doi.org/10.1039/D0GC04084C
13. Hernández M et al. Analysis of alkali-lignin in a paper mill effluent decolourised with two *Streptomyces* strains by gas chromatography-mass spectrometry after cupric oxide degradation. *J Chromatogr A*. 2001;**919**:389-94 https://doi.org/10.1016/s0021-9673(01)00813-5

14. Chen L et al. Comparative genome analysis of *Bacillus velezensis* reveals a potential for degrading lignocellulosic biomass. *3 Biotech*. 2018;**8**:253 https://doi.org/10.1007/s13205-018-1270-7

15. Khan AW, Tremblay D, LeDuy A. Assay of xylanase and xylosidase activities in bacterial and fungal cultures. *Enzyme Microb Technol*. 1986;**8**:373-77 https://doi.org/https://doi.org/10.1016/0141-0229(86)90139-0

16. Blondin B et al. Purification and properties of the β-glucosidase of a yeast capable of fermenting cellobiose to ethanol: *Dekkera intermedia* van der walt. *Eur J Appl Microbiol Biotechnol*. 1983;**17**:1-6 https://doi.org/10.1007/BF00510563

17. Chávez R, Bull P, Eyzaguirre J. The xylanolytic enzyme system from the genus *Penicillium*. *J Biotechnol*. 2006;**123**:413-33 https://doi.org/https://doi.org/10.1016/j.jbiotec.2005.12.036

18. Liu X et al. Acetylation of xenogeneic silencer H-NS regulates biofilm development through the nitrogen homeostasis regulator in *Shewanella*. *Nucleic Acids Res*. 2024;**52**:2886-903 https://doi.org/10.1093/nar/gkad1219

19. Biedka S et al. One-pot method for preparing DNA, RNA, and protein for multiomics analysis. *Commun Biol*. 2024;**7**:324 https://doi.org/10.1038/s42003-024-05993-1

20. Callahan BJ et al. DADA2: High-resolution sample inference from Illumina amplicon data. *Nat Methods*. 2016;**13**:581-3 https://doi.org/10.1038/nmeth.3869

21. Wu LY et al. Ubiquitous, B_{12}-dependent virioplankton utilizing ribonucleotide-triphosphate reductase demonstrate interseasonal dynamics and associate with a diverse range of bacterial hosts in the pelagic ocean. *ISME Commun*. 2023;**3**:108 https://doi.org/10.1038/s43705-023-00306-9

22. Bolger AM, Lohse M, Usadel B. Trimmomatic: a flexible trimmer for Illumina sequence data. *Bioinformatics*. 2014;**30**:2114-20 https://doi.org/10.1093/bioinformatics/btu170

23. Grabherr MG et al. Full-length transcriptome assembly from RNA-Seq data without a reference genome. *Nat Biotechnol*. 2011;**29**:644-52 https://doi.org/10.1038/nbt.1883

24. Zhang C et al. The majority of microorganisms in gas hydrate-bearing subseafloor sediments ferment macromolecules. *Microbiome*. 2023;**11**:37 https://doi.org/10.1186/s40168-023-01482-5

25. Hyatt D et al. Prodigal: prokaryotic gene recognition and translation initiation site identification. *BMC Bioinf*. 2010;**11**:119 https://doi.org/10.1186/1471-2105-11-119

26. Drula E et al. The carbohydrate-active enzyme database: functions and literature. *Nucleic Acids Res*. 2022;**50**:571-77 https://doi.org/10.1093/nar/gkab1045

27. Chen J et al. Metagenomic-based discovery and comparison of the lignin degrading potential of microbiomes in aquatic and terrestrial ecosystems via the LCdb database. *Mol Ecol Resour*. 2024;**24**:e13950

https://doi.org/10.1111/1755-0998.13950
28. Buchfink B, Xie C, Huson DH. Fast and sensitive protein alignment using DIAMOND. *Nat Methods*. 2015;**12**:59-60 https://doi.org/10.1038/nmeth.3176
29. Patro R et al. Salmon provides fast and bias-aware quantification of transcript expression. *Nat Methods*. 2017;**14**:417-19 https://doi.org/10.1038/nmeth.4197
30. Zhang ZF et al. Long-read assembled metagenomic approaches improve our understanding on metabolic potentials of microbial community in mangrove sediments. *Microbiome*. 2023;**11**:188 https://doi.org/10.1186/s40168-023-01630-x
31. Aroney STN et al. CoverM: read alignment statistics for metagenomics. *Bioinformatics*. 2025;**41**:btaf147 https://doi.org/10.1093/bioinformatics/btaf147
32. Song W et al. Functional traits resolve mechanisms governing the assembly and distribution of nitrogen-cycling microbial communities in the global ocean. *mBio*. 2022;**13**:e0383221 https://doi.org/10.1128/mbio.03832-21
33. Wood DE, Lu J, Langmead B. Improved metagenomic analysis with Kraken 2. *Genome Biol*. 2019;**20**:257 https://doi.org/10.1186/s13059-019-1891-0
34. Sayers EW et al. GenBank 2024 Update. *Nucleic Acids Res*. 2024;**52**:134-37 https://doi.org/10.1093/nar/gkad903
35. Mahmud B et al. Longitudinal dynamics of farmer and livestock nasal and faecal microbiomes and resistomes. *Nat Microbiol*. 2024;**9**:1007-20 https://doi.org/10.1038/s41564-024-01639-4
36. Du S et al. Divergent co-occurrence patterns and assembly processes structure the abundant and rare bacterial communities in a salt marsh ecosystem. *Appl Environ Microbiol*. 2020;**86**:e00322-20 https://doi.org/10.1128/aem.00322-20
37. Wang S et al. Temperature-Induced annual variation in microbial community changes and resulting metabolome shifts in a controlled fermentation system. *mSystems*. 2020;**5**:e00555-20 https://doi.org/10.1128/mSystems.00555-20
38. Knight CG et al. Soil microbiomes show consistent and predictable responses to extreme events. *Nature*. 2024;**636**:690-96 https://doi.org/10.1038/s41586-024-08185-3
39. Dhariwal A et al. Prolonged hospitalization signature and early antibiotic effects on the nasopharyngeal resistome in preterm infants. *Nat Commun*. 2024;**15**:6024 https://doi.org/10.1038/s41467-024-50433-7
40. Ning D et al. A quantitative framework reveals ecological drivers of grassland microbial community assembly in response to warming. *Nat Commun*. 2020;**11**:4717 https://doi.org/10.1038/s41467-020-18560-z
41. Brown JH et al. Toward a metabolic theory of ecology. *Ecology*. 2004;**85**:1771-89 https://doi.org/10.1890/03-9000
42. Zhou J et al. Temperature mediates continental-scale diversity of microbes in forest soils. *Nat Commun*. 2016;**7**:12083 https://doi.org/10.1038/ncomms12083
43. Zhou J et al. Stochastic assembly leads to alternative communities with distinct functions in a bioreactor microbial community. *mBio*.

2013;**4**:e00584-12 https://doi.org/10.1128/mBio.00584-12

44. Luo Y, Hui D, Zhang D. Elevated CO_2 stimulates net accumulations of carbon and nitrogen in land ecosystems: a meta-analysis. *Ecology*. 2006;**87**:53-63 https://doi.org/10.1890/04-1724
45. Yilmaz B et al. Microbial network disturbances in relapsing refractory Crohn's disease. *Nat Med*. 2019;**25**:323-36 https://doi.org/10.1038/s41591-018-0308-z
46. Miyauchi E et al. Gut microorganisms act together to exacerbate inflammation in spinal cords. *Nature*. 2020;**585**:102-06 https://doi.org/10.1038/s41586-020-2634-9
47. Hu Y et al. CAR-T cell therapy-related cytokine release syndrome and therapeutic response is modulated by the gut microbiome in hematologic malignancies. *Nat Commun*. 2022;**13**:5313 https://doi.org/10.1038/s41467-022-32960-3
48. Nottingham AT et al. Microbial diversity declines in warmed tropical soil and respiration rise exceed predictions as communities adapt. *Nat Microbiol*. 2022;**7**:1650-60 https://doi.org/10.1038/s41564-022-01200-1
49. Yehya N et al. Reappraisal of ventilator-free days in critical care research. *Am J Respir Crit Care Med*. 2019;**200**:828-36 https://doi.org/10.1164/rccm.201810-2050CP
50. Lynd LR et al. Microbial cellulose utilization: fundamentals and biotechnology. *Microbiol Mol Biol Rev*. 2002;**66**:506-77 https://doi.org/10.1128/mmbr.66.3.506-577.2002
51. Ahmed AAQ, Babalola OO, McKay T. Cellulase- and Xylanase-producing bacterial isolates with the ability to saccharify wheat straw and their potential use in the production of pharmaceuticals and chemicals from lignocellulosic materials. *Waste Biomass Valorization*. 2018;**9**:765-75 https://doi.org/10.1007/s12649-017-9849-5
52. Hage H, Rosso MN. Evolution of fungal Carbohydrate-Active Enzyme portfolios and adaptation to plant cell-wall polymers. *J Fungi*. 2021;**7**:185 https://doi.org/10.3390/jof7030185
53. Zheng LY et al. Oxygen dependent pyruvate oxidase expression and production in *Streptococcus sanguinis*. *Int J Oral Sci*. 2011;**3**:82-9 https://doi.org/10.4248/ijos11030
54. Ogola HJ et al. Molecular characterization of a novel peroxidase from the cyanobacterium *Anabaena* sp. strain PCC 7120. *Appl Environ Microbiol*. 2009;**75**:7509-18 https://doi.org/10.1128/aem.01121-09
55. Majeke BM et al. The synergistic application of quinone reductase and lignin peroxidase for the deconstruction of industrial (technical) lignins and analysis of the degraded lignin products. *Bioresour Technol*. 2021;**319**:124152 https://doi.org/10.1016/j.biortech.2020.124152
56. Alruwaili A, Rashid GMM, Bugg TDH. Application of *Rhodococcus jostii* RHA1 glycolate oxidase as an efficient accessory enzyme for lignin conversion by bacterial DyP peroxidase enzymes. *Green Chem*. 2023;**25**:3549-60 https://doi.org/10.1039/d3gc00475a

57. Pu Y, Cao S, Ragauskas AJ. Application of quantitative ^{31}P NMR in biomass lignin and biofuel precursors characterization. *Energy Environ Sci*. 2011;**4**:3154-66 https://doi.org/10.1039/C1EE01201K
